# Role of Intermolecular Interactions in Deep Eutectic Solvents for CO_2_ Capture: Vibrational Spectroscopy and Quantum Chemical Studies

**DOI:** 10.1021/acs.jpcb.4c04509

**Published:** 2024-10-09

**Authors:** Rashmi Mishra, Rajan Bhawnani, Rohan Sartape, Rohit Chauhan, Amey S. Thorat, Meenesh R. Singh, Jindal K. Shah

**Affiliations:** † School of Chemical Engineering, Oklahoma State University, 420 Engineering North, Stillwater, Oklahoma 74078, United States; ‡ Department of Chemical Engineering, University of Illinois at Chicago, 929 W. Taylor St., Chicago, Illinois 60607, United States

## Abstract

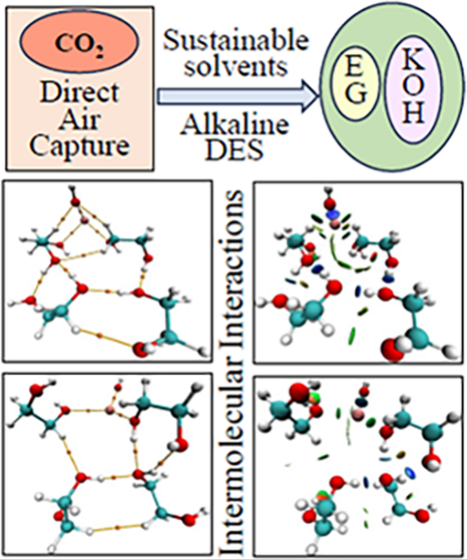

Recent research and reviews on CO_2_ capture methods, along with advancements in industry, have highlighted high costs and energy-intensive nature as the primary limitations of conventional direct air capture and storage (DACS) methods. In response to these challenges, deep eutectic solvents (DESs) have emerged as promising absorbents due to their scalability, selectivity, and lower environmental impact compared to other absorbents. However, the molecular origins of their enhanced thermal stability and selectivity for DAC applications have not been explored before. Therefore, the current study focuses on a comprehensive investigation into the molecular interactions within an alkaline DES composed of potassium hydroxide (KOH) and ethylene glycol (EG). Combining Fourier transform infrared (FT-IR) and quantum chemical calculations, the study reports structural changes and intermolecular interactions induced in EG upon addition of KOH and its implications on CO_2_ capture. Experimental and computational spectroscopic studies confirm the presence of noncovalent interactions (hydrogen bonds) within both EG and the KOH-EG system and point to the aggregation of ions at higher KOH concentrations. Additionally, molecular electrostatic potential (MESP) surface analysis, natural bond orbital (NBO) analysis, quantum theory of atoms-in-molecules (QTAIM) analysis, and reduced density gradient-noncovalent interaction (RDG-NCI) plot analysis elucidate changes in polarizability, charge distribution, hydrogen bond types, noncovalent interactions, and interaction strengths, respectively. Evaluation of explicit and hybrid models assesses their effectiveness in representing intermolecular interactions. This research enhances our understanding of molecular interactions in the KOH-EG system, which are essential for both the absorption and desorption of CO_2_. The study also aids in predicting and selecting DES components, optimizing their ratios with salts, and fine-tuning the properties of similar solvents and salts for enhanced CO_2_ capture efficiency.

## Introduction

According to reports from BBC, Future,^1^ reducing global warming by 1.5 °C in the next decade is crucial for protecting vulnerable ecosystems and communities. The concentration of CO_2_, one of the main contributors to global warming, is expected to reach 419.2 ppm this year, surpassing last year’s average of 417.2 ppm.^1^ Therefore, carbon dioxide removal (CDR) technologies are essential for achieving net-zero emissions and reducing atmospheric current and historical emissions.^2^ Direct air capture (DAC) is a promising CDR method that extracts CO_2_ from the air. It is stored underground or used in various applications, providing verifiable, permanent CO_2_ removal and making it vital to achieving net-zero emissions.^3−6^ Despite its potential, DAC faces significant cost challenges due to the energy-intensive process of capturing CO_2_ from low concentrations in the air.^7^ The U.S. government is investing significantly in DAC to overcome these obstacles and scale the technology.^3,8^

DAC technologies are categorized into solid DAC (S-DAC) and liquid DAC (L-DAC). S-DAC uses solid sorbents to adsorb or absorb CO_2_, requiring temperature or pressure swings for capture and release, which can be energy-intensive and complex.^5,9^ In contrast, L-DAC utilizes liquid solvents, leveraging chemical reactions for efficient CO_2_ capture, offering better selectivity, flexibility, and scalability, and benefiting from existing liquid-based CO_2_ capture infrastructure.^5^ Traditional aqueous amine solvents, such as monoethanolamine (MEA), are commonly used in L-DAC but suffer from high energy consumption, solvent loss, and environmental toxicity.^10^ Ionic liquids (ILs) are potential substitutes due to their low vapor pressure, high thermal stability, and strong solubility.^11,12^ However, they can be expensive and complex to synthesize, and many ILs face biodegradability and toxicity issues.^13,14^ Therefore, deep eutectic solvents (DESs) have emerged as a promising alternative for CO_2_ capture and are more eco-friendly and cheaper than traditional methods.^15,16^

DESs are solutions of Lewis or Brønsted acids and bases, comprising hydrogen bond donors (HBDs) and acceptors (HBAs).^16,17^ They share many properties with ILs, such as low vapor pressure, high thermal stability, and low volatility, but are cheaper, biodegradable, and easier to prepare.^17−19^ By adjusting their components and molar ratios, DESs can be modified for high CO_2_, solubility, and selectivity.^20,21^ Reactive DESs contain an HBA, a quaternary ammonium salt, or choline chloride, and an HBD, such as urea, carboxylic acids, or polyols like glycerol. These components have functional groups that chemically react with CO_2_ to form carbamates. While effective in binding CO_2_, reactive DESs may degrade over time or under operational conditions. In contrast, alkaline DESs typically consist of an alkaline salt, such as potassium hydroxide or lithium chloride as the HBA, combined with HBDs such as urea, glycerol, or ethylene glycol. These combinations impart basicity to the solvent system, facilitating the formation of bicarbonates, which are more stable than carbamates.^22^ Additional advantage of this reaction is that it does not lead to a dramatic increase in the viscosity of the system as has been found when carbamates are formed.^23^

The efficiency of CO_2_ absorption in DESs depends on the interactions between HBA and HBD because they directly influence the physical and chemical properties of the solvent. These interactions include electrostatics, hydrogen bonding (H-bonding), van der Waals forces, induction, dispersion, and exchange counterparts. Understanding these molecular interactions is crucial because they must be stable to enhance the solubility of CO_2_ within the solvent and contribute to maintaining the solvent structure while accommodating CO_2_ molecules and reversible, offering easier regeneration of solvent and CO_2_. We chose an alkaline DES of HBA as potassium hydroxide (KOH) and HBD as ethylene glycol (EG) to analyze interaction patterns.^24−26^ KOH is an attractive choice in industrial CO_2_ removal due to its cost-effectiveness, high absorption capacity, and stability against degradation.^25−27^ EG is a renewable, commonly used HBD polar solvent suitable for CO_2_ capture^26,28,29^ and is a high-boiling-point liquid, minimizing solvent losses. Additionally, organic solutions containing KOH and EG are used as cost-effective transition temperature mixtures (TTMs) for nonaqueous and reversible CO_2_ sorption.^26,30^ Electrochemical CO_2_ capture methods employing a migration-assisted moisture gradient (MAMG) technique also utilize KOH and EG.^30^ The molar ratio of components in DESs plays a crucial role in determining their performance of CO_2_ solubility.^31^ Higher KOH concentrations increase DES basicity, aiding CO_2_ absorption by forming bicarbonate ions. However, a too high concentration can increase the viscosity, hinder CO_2_ diffusion, and also decrease the CO_2_ solubility due to the salting-out effect.^32−34^ Therefore, the impact of the KOH concentration on intermolecular interactions is a critical factor, which will be analyzed.

The current study integrates quantum chemical (QC) studies employing density functional theory (DFT)^35^ with vibrational spectroscopy to comprehensively understand molecular structures and intermolecular interactions.^36−40^ By comparing the results of DFT calculations with experimental spectroscopic data, we analyzed changes in FT-IR spectra, focusing on peaks and additional modes introduced in EG after adding KOH.^36−40^ Visualization of interaction sites and changes in polarizability was performed using molecular electrostatic potential (MESP) surface analysis.^41,42^ Natural bond orbital (NBO) analysis provided insights into charge distribution and type of hydrogen bonds within the EG-KOH system.^43^ Topological analysis using the quantum theory of atoms-in-molecules (QTAIM)^44,45^ elucidated the nature and strength of noncovalent interactions, further dissected through reduced density gradient-noncovalent interaction (RDG-NCI)^46^ plot analysis. A comparison between explicit and hybrid models was conducted to assess which model faithfully represents the intermolecular interactions. This comprehensive computational approach enabled a detailed investigation of the molecular properties, electronic structure, chemical reactivity, and noncovalent interactions. Understanding how different interactions influence EG’s properties after adding KOH enhances the prediction and optimization of DES components, improving CO_2_ capture efficiency for DAC technologies.

## Experimental Details

### Materials and Sample Preparation

EG (99% purity) and KOH (99.99% purity) pellets were obtained from Sigma-Aldrich. Prior to use in an experiment, EG was dried in a vacuum oven at 60 °C for 12 h. The water content of EG was determined to be less than 0.1% using a Metrohm Karl Fisher Titrator. For the preparation of the KOH and EG samples at various concentrations, calculated amounts of KOH were weighed on an analytical weighing balance (Accuris W3100-210) and later dissolved in EG using a magnetic stirring setup at 250 rpm for 3 h.

### Fourier Transform Infrared Spectroscopy (FT-IR)

The infrared spectra of the freshly prepared samples were recorded on a Bruker Invenio-S spectrometer in attenuated total reflectance (ATR) mode using a Pike VeeMax-III ATR module. A ZnSe crystal fixed on a steel base was also placed on the ATR accessory. A 20 μL portion of the prepared sample was dispensed, using a micropipet, every time on a ZnSe crystal for recording the spectra. The spectrum at each time was recorded with a spectral resolution of 2 cm^–1^, averaging 32 scans. The beam aperture was set to 6 mm, and the specular angle was set to 60°. All spectra were recorded under standard atmospheric pressure and room temperature.

## Theoretical Details

In the present study, electronic structure calculations for pure ethylene glycol and mixtures of KOH-EG were carried out. A minimum-energy geometry was obtained for a single EG molecule in the gas phase. Additionally, geometries of dimer and tetramer were optimized in the gas phase in a continuum solvation model of the dielectric constant of 37 consistent with that of EG from the starting configurations obtained using Packmol.^47^ Similarly, several ratios of KOH/EG were probed: 1:1, 1:2, and 1:4 in the gas phase as well as employing a continuum solvation model at the B3LYP/6-311++G(d,p) level^35,48^ using Gaussian 09.^49^ The B3LYP functional and 6-311++G(d,p) basis set were chosen for their accuracy and computational efficiency in studying hydrogen-bonded organic complexes.^50,51^ This widely used combination ensures reliable geometries, energies, and vibrational frequencies, despite B3LYP’s known limitations in describing van der Waals interactions.^52−55^ It provides a cost-effective starting point for modeling medium-sized organic molecules and is particularly useful in hybrid models where computational efficiency is crucial.^56,57^

Vibrational spectra calculated from these structures were compared to those measured experimentally. DFT calculations often overestimate the computed frequencies of vibrational modes compared to observed values due to neglect of the anharmonicity present in a real system. To compensate for this discrepancy, the calculated frequencies were scaled using the wavenumber-linear scaling (WLS) procedure.^58,59^ The WLS procedure enhances the accuracy of vibrational frequencies by applying different scaling factors for various frequency ranges based on the linear function of the wavenumber. Unlike uniform scaling, which uses a single factor for all frequencies, the WLS employs distinct scaling factors for different frequency ranges. Commonly used with DFT methods like B3LYP, WLS can reduce mean absolute errors by 20–30% compared to uniform scaling.^58−60^ The WLS formula used is shown below:
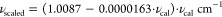
1The Vibrational Energy Distribution Analysis (VEDA) program^61^ was used for assignments of vibrational frequencies based on potential energy distribution (PED) values.

The stability of a given KOH-EG complex was quantified by computing interaction energy (IE) using the following expression:
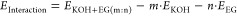
2where *m* is the number of KOH molecules and *n* represents the number of EG molecules. The chemical hardness (η)^62,63^ was calculated using the energies of highest occupied molecular orbital (*E*_HOMO_) and energies of lowest unoccupied molecular orbital (*E*_LUMO_) using the following expression:
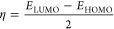
3The interactions were assessed using NBO analysis.^64,65^ QTAIM graphs and RDGs-NCI isosurfaces were calculated using the Multiwfn 3.7 program.^66^ The stabilization energy *E*^(2)^ was calculated using second-order Fock matrix analysis
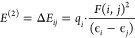
4where *q*_*i*_ is the donor orbital occupancy, ϵ_*i*_ and ϵ_*j*_ are diagonal elements’ energies, respectively, and *F*(*i*, *j*) is the off-diagonal NBO Fock matrix element.^67^ The strength of a given hydrogen bond (H-bond)^68^ was calculated using the following expression:
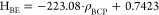
5where ρ_BCP_ is the electron density and is expressed in atomic units, while the H-bond energy is in kcal/mol.

We considered two primary approaches to assess solvent effects: explicit and hybrid solvent models.^69^ In the explicit (atomistic) solvent model, the system includes explicitly modeled solvent molecules surrounding the solute^70^ accounting for short-range interactions such as H-bonding and polarization. Meanwhile, in implicit (continuum) approaches, solvent molecules are replaced with a homogeneously polarizable medium.^71^ However, the implicit model may lack short-range interactions (H-bonds, etc.), potentially leading to inaccuracies in evaluating solute–solvent effects.^72^ Therefore, for a thorough analysis of intermolecular interactions in the solvent phase, a hybrid model (a combination of explicit and implicit) was utilized.^73^ For the solvent effects, the integral equation formalism polarizable Continuum model (IEFPCM) was employed, treating the solvent EG as a continuous dielectric medium with averaged properties and a dielectric constant of 37 to capture the effects of solute–solvent interactions.^74^ In the case of hybrid solvation models, the ratios mentioned throughout the article indicate the number of KOH and EG molecules modeled explicitly. For example, a KOH-EG ratio of 1:2 in a hybrid model implies one KOH molecule and two EG molecules modeled explicitly.

## Results and Discussion

### Inferences of Experimental Data

**Figure 1:**
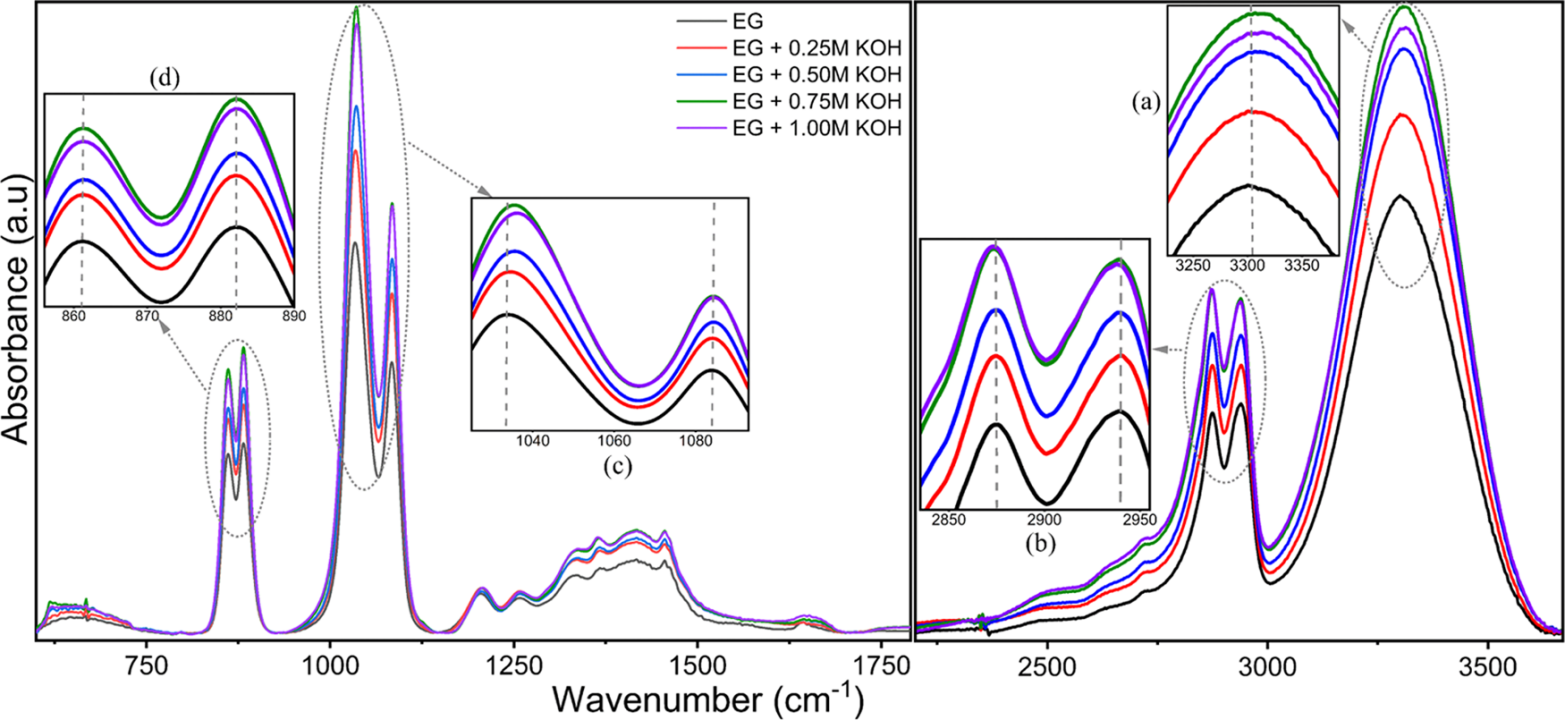
Comparison of experimental FT-IR spectra of EG and KOH-EG complexes for KOH concentrations of 0.25, 0.50, 0.75, and 1 M along with magnified versions for the following frequency regions: (a) 3220–3385 cm^–1^, (b) 2830–2950 cm^–1^, (c) 1025–1090 cm^–1^, and (d) 850–890 cm^–1^.

The H-bonding network influences the viscosity of the DES, which is an essential factor in deciding how easily CO_2_ can diffuse through the solution before reacting.^21,75^ Experimental techniques, such as Fourier transform infrared spectroscopy (FT-IR), provide valuable insights into H-bonding. Therefore, the FT-IR spectra for EG and KOH-EG complexes, recorded after adding 0.25, 0.50, 0.75, and 1 M of KOH to EG, are compared and presented in Figure 1. The comparison of frequencies and full width at half-maximum (FWHM) for the prominent peaks at all molar ratios is provided in Table S1a,b in the Supporting Information (SI).

#### Blue Shift of OH Stretching

In the spectrum of EG, a broad peak is observed in the OH stretching frequency region (3000–3600 cm^–1^), indicating that hydroxyl groups are involved in H-bonding. On comparing the FT-IR spectra of EG and KOH-EG complexes, peak broadening and an increase in intensity are observed in the higher-frequency region with increasing KOH concentration in EG. To better view the peak position of OH stretching, the frequency region 3220–3385 cm^–1^ of the spectra is magnified and presented in Figure 1a, in which the dotted vertical line represents the wavenumber at which the maximum in the spectra is observed for pure EG. A small blue shift is observed for the wavenumber as KOH is added to the EG, indicating a change in H-bonding between EG molecules. The blue shift in the recorded IR spectra signifies that the bond length in OH is shortened in the presence of KOH, which is a consequence of two opposing factors: hyperconjugative interaction and hybridization/polarization effects. Hyperconjugation, involving electron density transfer from the antibonding orbital of O–H, typically elongates the H-bond, causing a red shift in its IR stretching frequency as in the case of proper H-bonds. Conversely, the polarization of the H-bond between OH^–^ and hydrogen of the OH group in EG leads to a reduction in the H-bond length, which results in a blue shift in the stretching frequency. These H-bonds are termed improper H-bonds.^76^ In addition, the negatively charged OH^–^ ion (hydrogen bond acceptor) and the electron-deficient hydrogen in EG (hydrogen bond donor) lead to an ion-dipole interaction, contributing to a stronger H-bonding interaction and reduction in H-bond length in comparison to pure EG. It is also observed that the blue shift slightly increases when the concentration of KOH progressively increases from 0.25 to 0.75 M. However, the blue shift is nearly identical for 0.75 and 1 M. This indicates a saturation in H-bonding with increasing KOH concentrations.

#### OH Peak Broadening

In the case of KOH-EG complexes, peak broadening is also observed in the 3000–3600 cm^–1^ range, which suggests multiple overlaps of vibrational modes. These arise due to an increase in the intermolecular interactions as KOH provides additional interaction sites for H-bonding, ion-dipole interactions, dipole–dipole interactions, and electrostatic interactions. Peak broadening also reveals that the addition of KOH increases the type of H-bonded interactions in the system.

#### OH Peak Intensification

Furthermore, the intensities of the maximum also amplify as the KOH concentrations increase due to the formation of improper H-bonds in addition to proper H-bonding observed in pure EG. However, this effect reverses when the concentration of KOH increases from 0.75 to 1.0 M which can be attributed to the aggregation of K^+^ and OH^–^ as observed by Thorat et al.;^77^ such aggregation would result in the reduction of H-bonding opportunities of OH^–^ with EG. This signifies that the extent of peak broadening and intensity depends on the concentration of KOH and EG and the strength and type of intermolecular interactions.

#### Effect of OH Modulations on C–H, C–C, and C–O Vibrational Modes of EG

On comparing the frequency region of CH stretching (2800–3000 cm^–1^) it is observed that the peak intensity increases with increasing KOH concentration; however, the peaks for 0.75 and 1 M KOH overlap. The increase in intensity is again because of more H-bond formation after adding KOH to EG and then attaining saturation for higher KOH ratios. A red shift in the peaks for CH stretching, observed in the magnified version (2830–2950 cm^–1^) as shown in Figure 1b, again confirms improper H-bond formation between the OH^–^ ion and hydroxyl groups of EG. H-bonding interactions involving the OH^–^ ion reduce the electron density in the C–O bond, leading to a lower vibrational frequency due to a less rigid bond, hence a red shift of CH stretching frequencies in the case of improper H-bonds. On analyzing the backbone C–C and C–O stretching vibration modes in the frequency region 1000–1100 cm^–1^, an increase in intensity and width is observed for lower KOH ratios, with saturation occurring at higher KOH ratios. In the magnified version (1025–1090 cm^–1^) shown in Figure 1c, a blue shift is observed in the C–O peak on the left, which again supports improper H-bond formation after adding KOH to EG. On comparing the frequency region 825–925 cm^–1^ of C–C and C–O stretching and CH_2_ bending vibration modes and observing the magnified version (825–925 cm^–1^) shown in Figure 1d, the same increase in intensity with an increase in the KOH ratio and saturation at higher KOH ratios is observed.

The observed shifts in the FT-IR spectra indicate the presence of improper H-bonds in the KOH-EG system.^78^ These nonconventional H-bonds significantly affect the mixture’s molecular packing, viscosity, and molecular mobility, influencing CO_2_ diffusion and reaction kinetics during absorption and desorption.^79^ Optimizing these H-bonds by adjusting the KOH-EG ratio or adding components can enhance CO_2_ capture systems by improving selectivity, accelerating absorption/desorption kinetics, and reducing regeneration energy requirements.^79−82^ However, the impact of these interactions is dependent on the specific composition and thermodynamic conditions of the KOH-EG mixture, requiring a comprehensive analysis across various parameters.

The results mentioned above validate the existence of H-bonding between OH^–^ and EG, which not only stabilizes the DES (KOH-EG complex) but also facilitates an interactive solvent environment,^83^ dictating the physical state of DES and influencing their ability to absorb CO_2_.^21,75,84^ Furthermore, on increasing the concentration of KOH beyond 0.75 M could potentially reduce CO_2_ absorption because higher concentrations of KOH may lead to aggregation, thereby elevating viscosity and decreasing CO_2_ diffusion rates^83^ as indicated by saturation at 1 M KOH. Additionally, aggregation may limit the availability of free, active OH^–^ ions crucial for CO_2_ interaction, resembling the salting-out effect.^32−34^ This effect arises as the KOH concentration increases, raising the ionic strength of the KOH-EG solution, disrupting the solvation layer around EG molecules, and reducing KOH solubility. The existing OH^–^ ions bind with EG molecules, decreasing the availability of EG to interact with additional OH^–^ ions introduced with increased KOH concentration. This competition further reduces the solubility of KOH. As KOH solubility decreases, K^+^ and OH^–^ ions, once dissolved in EG, may aggregate and precipitate, causing KOH to “salt out” from the solution. The salting-out ratio is crucial for solvent regeneration, helping determine the concentration at KOH precipitate due to increased ionic strength, guiding effective solute removal for solvent reuse.^32^ Hence, selecting an appropriate KOH concentration is crucial, and 0.75 M could be suitable.

#### Interactions beyond H-Bonding

The FT-IR spectra provide insights into H-bonding but offer no information about other intermolecular interactions. van der Waals interactions, such as London dispersion forces, arising from fluctuations in electron density, can also lead to peak broadening; this effect is less pronounced than H-bonding. van der Waals interactions also influence the viscosity and density, which in turn affect CO_2_ diffusion and absorption.^21,83^ Relatively small red shifts (<100 cm^–1^) can result from hyperconjugative effects in the diol skeleton, especially without intramolecular H-bonding.^85^ The FT-IR spectra suggest an increase in polarizability, but changes in polarizability can also impact peak width due to alterations in the molecular charge distribution affecting vibrational modes. Additionally, peak broadening can result from factors such as changes in the temperature, sample concentration, solvent effects, and instrumental limitations. Peak broadening alone may not be sufficient to determine the nature of the interactions.^85^ Therefore, it is necessary to supplement FT-IR spectral observations with theoretical calculations and analysis to gain a comprehensive understanding of the intermolecular interactions (van der Waals, steric interactions, etc.) and changes in polarizability.

### Results from DFT Calculations

#### Geometric Configuration of EG and KOH-EG Mixture

**Figure 2:**
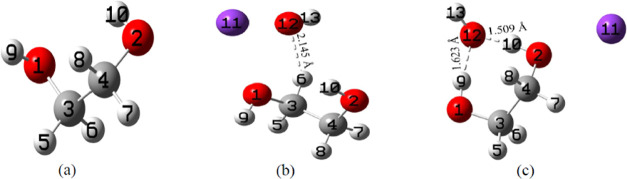
Optimized geometries of (a) the monomer, (b) the KOH+EG 1:1 ratio explicit model, and (c) the KOH+EG 1:1 ratio hybrid model. Gray, red, white, and purple colors represent C, O, H, and K atoms, respectively, and dotted lines denote H-bonds. (b, c) H-bonds formed after adding KOH to EG.

**Figure 3:**
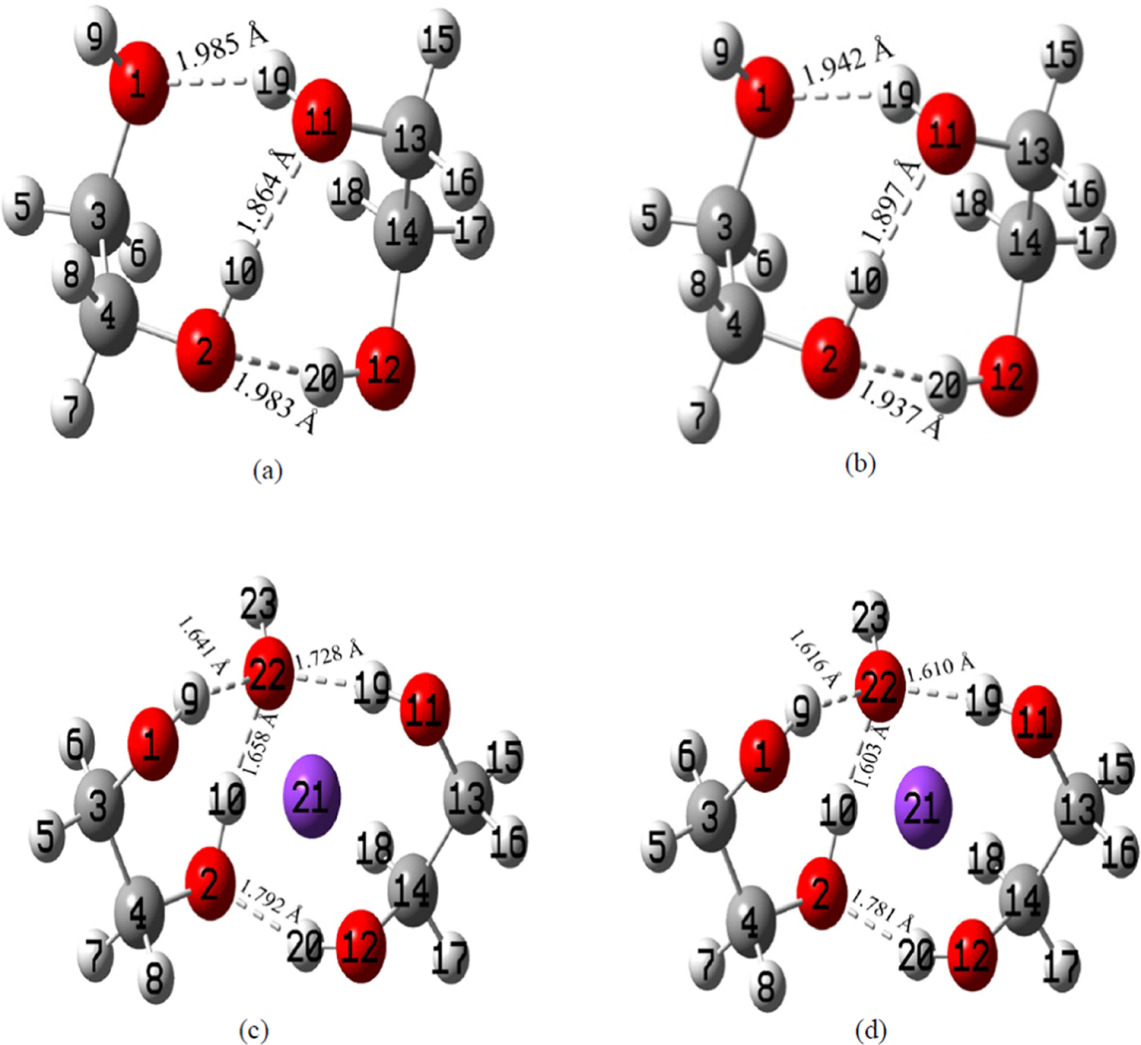
Optimized geometries of (a) the EG dimer explicit model, (b) the EG dimer hybrid model, (c) the KOH+EG 1:2 ratio explicit model, and (d) the KOH+EG 1:2 ratio hybrid model. Gray, red, white, and purple colors represent C, O, H, and K atoms, respectively, and dotted lines denote H-bonds. (a, b) H-bonds in the EG dimer and (c, d) newly formed H-bonds and the changes in the existing H-bonds of the EG dimer following the addition of KOH to EG.

**Figure 4:**
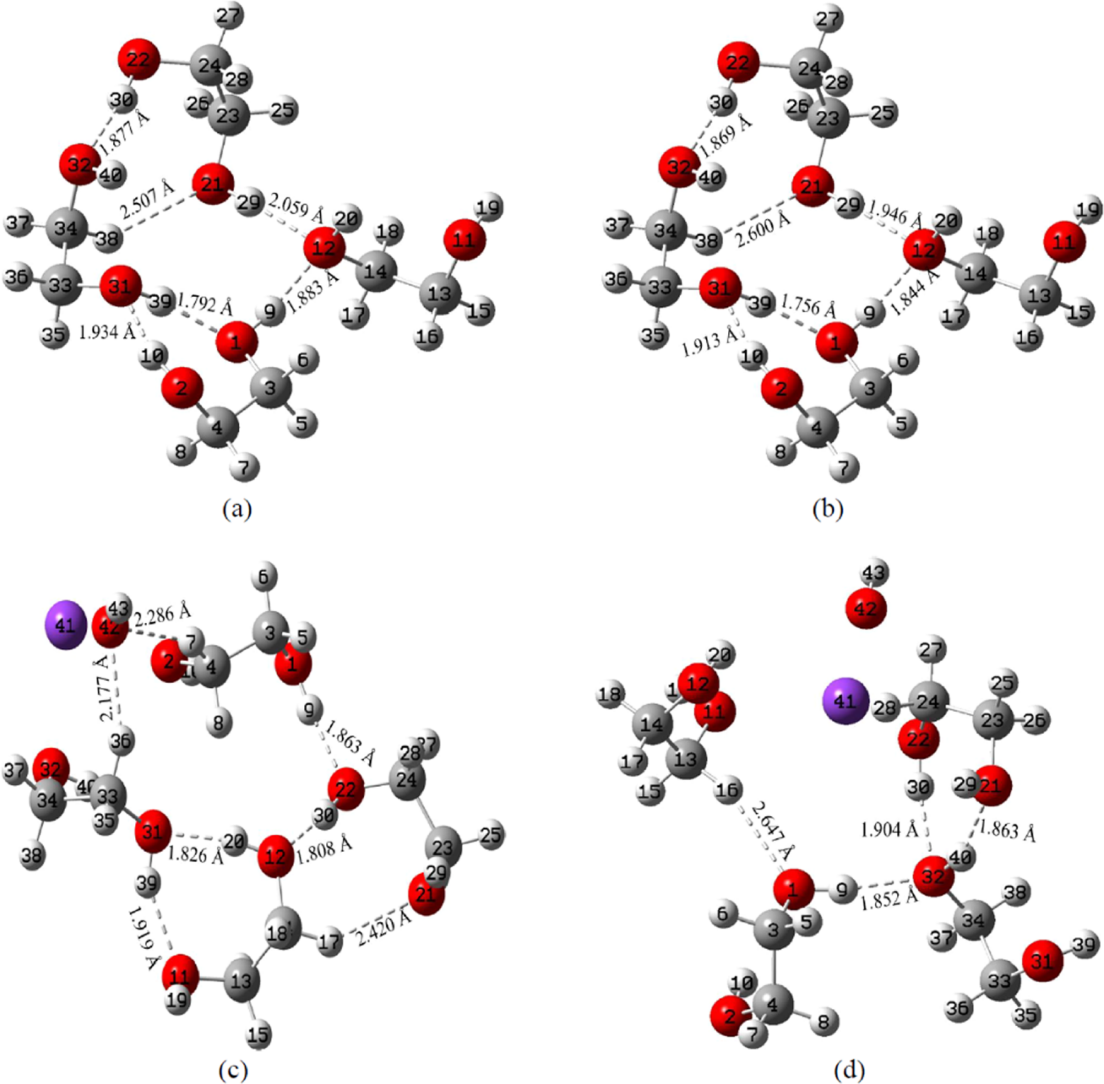
Optimized geometries of (a) the EG tetramer explicit model, (b) the EG tetramer hybrid model, (c) the KOH+EG 1:4 ratio explicit model, and (d) the KOH+EG 1:4 ratio hybrid model. Gray, red, white, and purple colors represent C, O, H, and K atoms, respectively, and dotted lines denote H-bonds. (a, b) H-bonds in the EG tetramer and (c, d) changes (newly formed and broken) in the existing H-bonds of the EG tetramer following the addition of KOH to EG.

Geometry optimization was performed to understand the molecular structures, forming the basis for further analysis. Gauche conformation in EG dominates at room temperature,^85−87^ and was utilized for building EG oligomers and KOH-EG complexes. The gas-phase optimized geometries are portrayed in Figure 2a for the monomer, in Figure 3a for the dimer, and in Figure 4a for the tetramer. The corresponding geometries obtained in the presence of a continuum solvation model are depicted in Figures 3b and 4b for the dimer and tetramer, respectively. Tables S2 and S3 in the SI give the corresponding PDB files. The optimized geometry of KOH-EG complexes shows that the bonds between the EG oligomers are changed after the addition of KOH due to alterations resulting from the interaction of hydroxyl groups of EG with the potassium (K^+^) and hydroxide (OH^–^) ions.

#### Distribution of H-Bonds

The van der Waals (VDW) radii provide a reference for identifying H-bonds within molecular structures. Using the VDW radii of 1.52 and 1.20 Å for oxygen and hydrogen, resepctively,^88^ intermolecular bonds shorter than 2.72 Å (sum of the two VDW radii) signal the presence of H-bonds. The H-bonds are marked as dotted lines, and the values are given in the optimized structures. The visualization of the structures conveys the existence of several intermolecular H-bonding interactions and the absence of intramolecular H-bonds. This explains the reason behind the retention of gauche conformation because of hyperconjugation effects between the C–H and O–H groups in the absence of intramolecular H-bonding and is in confirmation with earlier results.^85^

After adding KOH to EG, changes in the H-bond lengths, the disappearance of certain existing H-bonds, and the formation of some new bonds are noticed (refer to Figures 2a,b, 3c,d, and 4c,d). This suggests that K^+^ and OH^–^ ions disrupt some of the H-bonding interactions in EG, likely through ion-dipole interactions with polar regions of EG. Some C–H–O, H-bonds are also observed for tetramer and some KOH-EG complexes, which are in confirmation with earlier experimental results.^85,89,90^ The difference in H-bond lengths between explicit and hybrid solvent models can be attributed mainly to the different treatments of solvation effects. The differences are more prominent for the KOH-EG complexes. Hybrid models integrate explicit representation of EG molecules with an implicit continuum treatment of the bulk EG solvent, resulting in a more accurate representation of H-bonding compared to explicit models. These differences highlight the importance of considering both local and bulk solvation effects for accurately modeling intermolecular interactions in solution.

The propensity for the formation of the KOH-EG complex can be discerned from the interaction energy which is recorded in Table 1. For pure EG, the interaction energy is found to be negative for dimers and tetramers, indicating the stability and existence of these structures in liquid EG. Similarly, the negative interaction energy upon the addition of KOH is an indicator that the mixing process is driven by enthalpic contributions, the origin of which can be traced to the formation of multiple H-bonding interactions between OH^–^ and EG. The interaction energy was found to depend on the number of EG molecules explicitly used in the calculation. Despite the variation, the negative values indicate that the solvation of KOH is energetically favorable.

**Table 1:** Comparison of the Interaction Energy (IE) of EG Oligomers with KOH-EG Complexes

	IE (kcal/mol)			IE (kcal/mol)	
EG	explicit	hybrid	KOH-EG complexes	explicit	hybrid
			1:1 ratio	–14.05	–19.159
dimer	–13.229	–9.772	1:2 ratio	–59.736	–35.190
tetramer	–30.371	–23.125	1:4 ratio	–54.450	–24.022

#### CO_2_ Reactivity Analysis from HOMO–LUMO

The ability of the KOH-EG complex to interact with CO_2_ depends on the energy difference between its HOMO and LUMO of CO_2_. The reduction in the HOMO level of the KOH-EG complex, as demonstrated in Table 2 when KOH is added to EG, decreases the energy gap between its HOMO and the LUMO of CO_2_. This reduction can potentially facilitate efficient electron transfer from the HOMO of the KOH-EG complex to the LUMO of CO_2_, thus enhancing CO_2_ capture. Moreover, the decrease in the bandgap, as evidenced in Table 2, on adding KOH to EG suggests increased reactivity of the KOH-EG complex in comparison to EG. This increased reactivity also indicates the possibility of enhanced charge transfer and interactions of the KOH-EG complex with CO_2_ molecules, potentially leading to higher absorption rates and capacities.

**Table 2:** Comparison of HOMO, LUMO Energies, and Bandgaps of EG Oligomers, KOH-EG Complexes, and CO_2_^a^

			EG					
	CO_2_		monomer		dimer		tetramer	
parameters (eV)	EXC	HYB	EXC	HYB	EXC	HYB	EXC	HYB
HOMO	–10.499	–10.401	–7.581	–7.648	–7.142	–7.401	–6.790	–7.163
LUMO	–0.537	–0.347	–0.569	–0.279	–0.746	–0.309	–1.126	–0.470
bandgap	9.962	10.054	7.013	7.369	6.396	7.092	5.664	6.692

			KOH-EG complexes					
			1:1 ratio		1:2 ratio		1:4 ratio	
parameters (eV)			EXC	HYB	EXC	HYB	EXC	HYB
HOMO			–4.825	–6.359	–6.829	–6.896	–4.696	–5.842
LUMO			–1.082	–0.432	–0.7412	–0.397	–0.789	–0.598
bandgap			3.743	5.927	6.088	6.499	3.908	5.244

a EXC refers to the explicit representation while HYB denotes the presence of a continuum solvation model.

#### Deconvolution and Validation of Molecular Interaction by Predictive Simulation of IR Spectra

**Figure 5:**
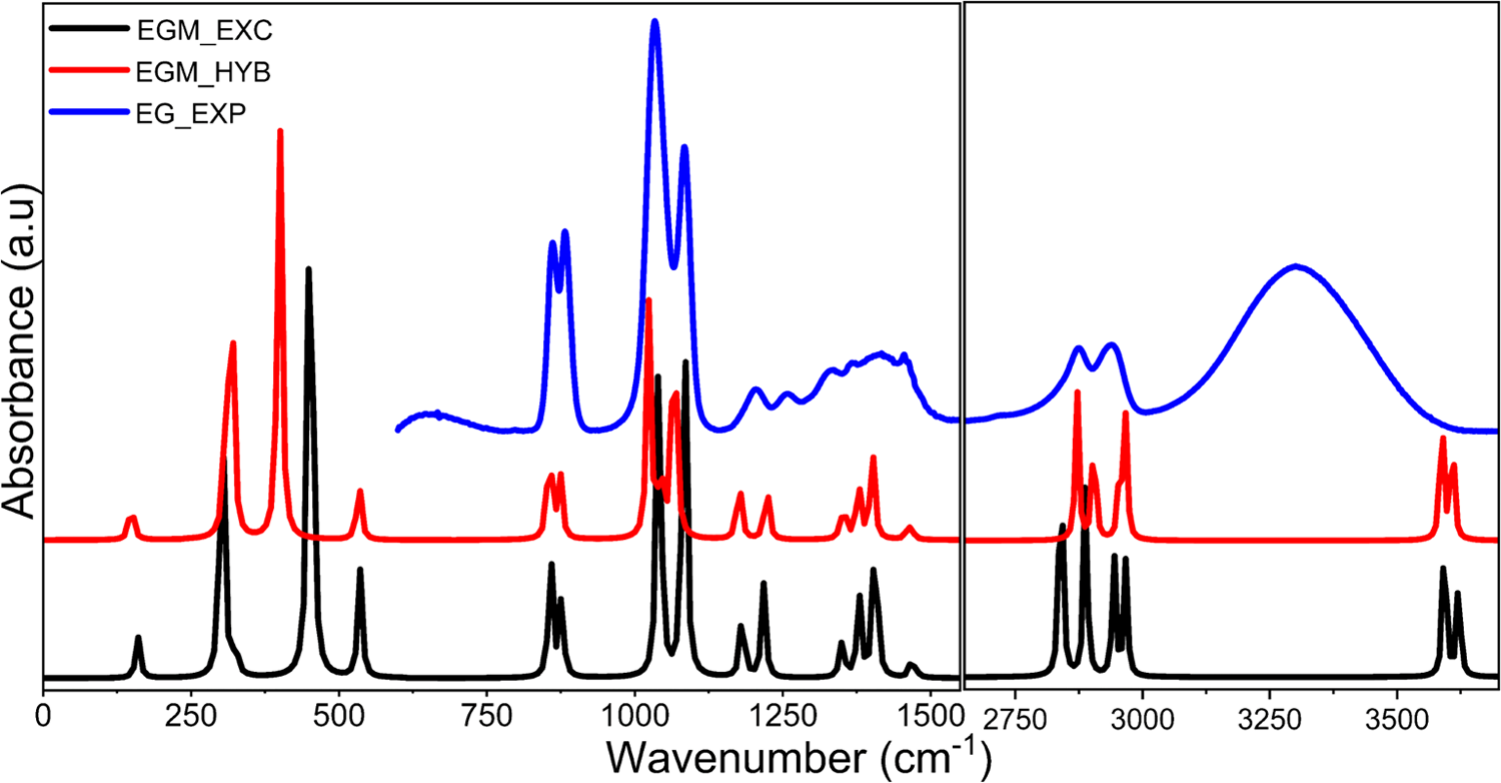
Comparison of experimental (EXP) and calculated IR spectra of the ethylene glycol monomer (EGM) for both explicit (EXC) and hybrid models.

**Figure 6:**
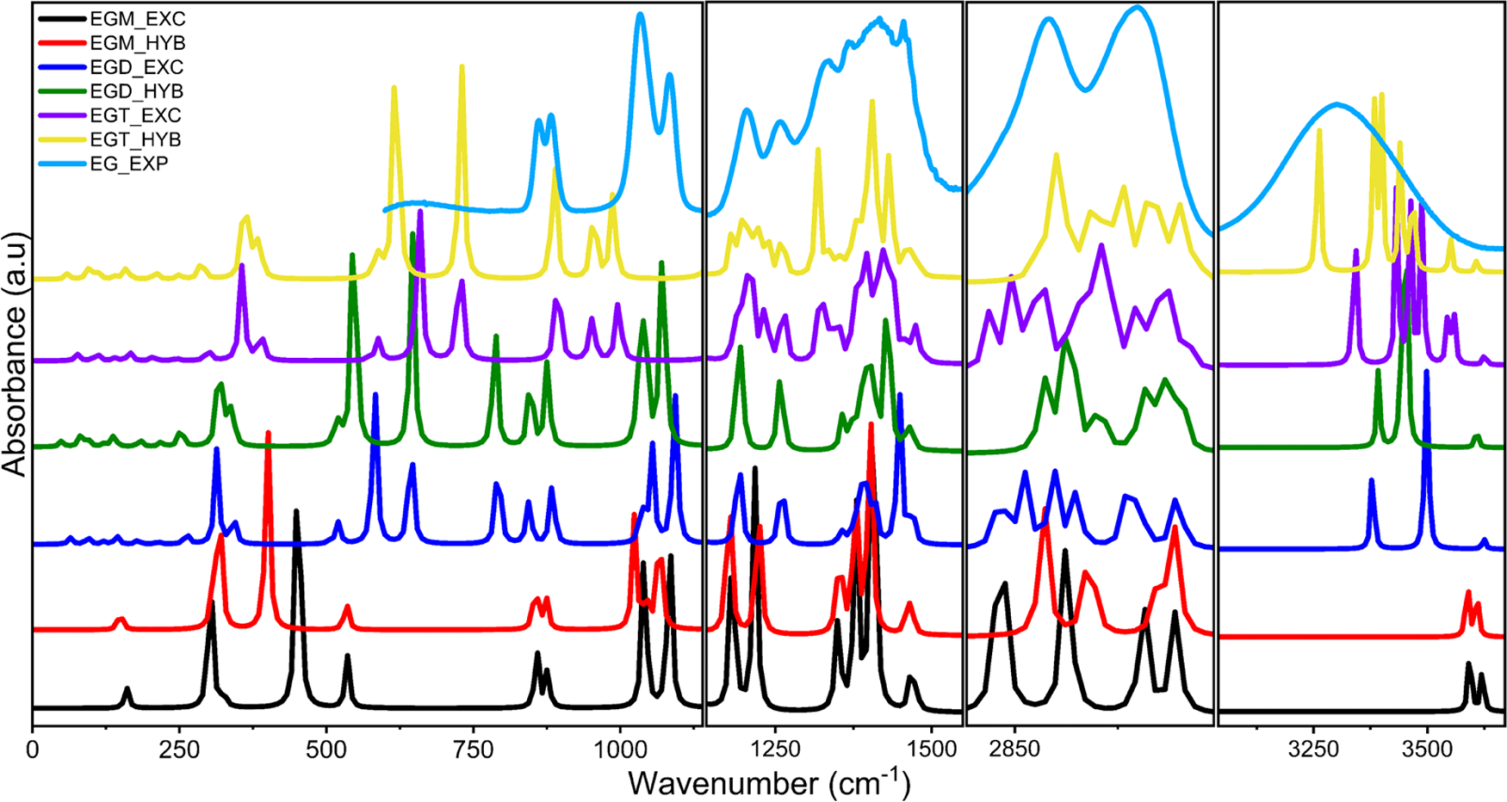
Comparison of IR spectra of experimental (EXP) and theoretical ethylene glycol monomer (EGM), dimer (EGD), and tetramer (EGT) for both explicit (EXC) and hybrid (HYB) models.

To identify the exact nature of interactions in KOH-EG systems, we analyze vibrational spectra obtained from quantum calculations outlined earlier for geometry optimization. In this work, a comparison between the calculated and experimental spectra is presented in Figure 5, while the vibrational modes obtained from PED are provided in Table S4 in the SI. The calculated EG monomer spectra agree well with the experimental spectra for lower-frequency regions (750–1500 cm^–1^). However, calculated EG monomer spectra differ significantly in the OH stretching frequency range (3000–3600 cm^–1^) and are marked by the absence of a broad peak observed experimentally. The higher-frequency region contains information about the vibrational mode corresponding to OH stretching, which is sensitive to intermolecular interactions. As the calculation is performed only for a monomer, these interactions cannot be captured. We carried out calculations on the EG dimer and tetramer to incorporate these effects. A comparison of calculated (explicit and hybrid) spectra for EG oligomers and experimental spectra is shown in Figure 6, while the assignment of vibrational modes is presented in Table S4 in the SI. Figure 6 shows that the number of OH stretching peaks increases as we increase the number of EG units due to an increase in H-bonding. In the case of the tetramer, many OH stretching peaks, when combined, could be correlated to the broad peak in the experimental spectra in the higher-frequency region. A red shift is perceived for OH stretching, and a blue shift is perceived for CH stretching modes when moving from monomer to dimer and dimer to tetramer for both explicit and hybrid models as analyzed in Table S4. These shifts indicate the formation of H-bonds. A prominent red shift is seen for hybrid models rather than explicit models because the hybrid model can capture the interaction of hydroxyl groups of EG through H-bonding and other intermolecular forces with the EG solvent environment, mimicking the experimental system as it can capture both the interactions among the molecules and the solvent environment. Since the hybrid tetramer model matches the experimental spectra better, higher units and hybrid models can capture the interactions better for spectra representation and analysis.

**Figure 7:**
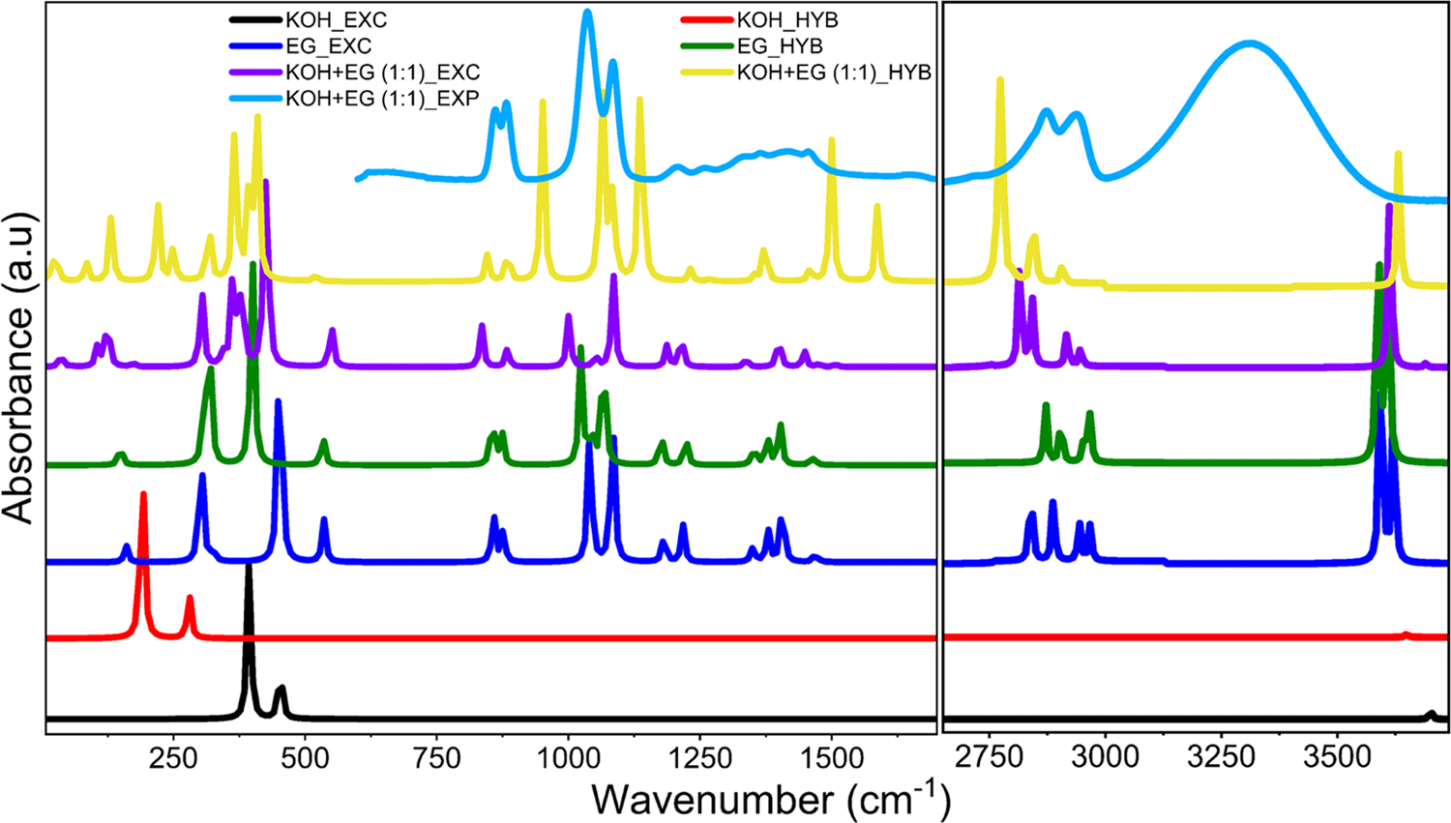
Comparison of IR spectra of calculated KOH, EG, and KOH+EG 1:1 ratio with experimental (EXP) EG+KOH spectra for both explicit (EXC) and hybrid (HYB) models.

**Figure 8:**
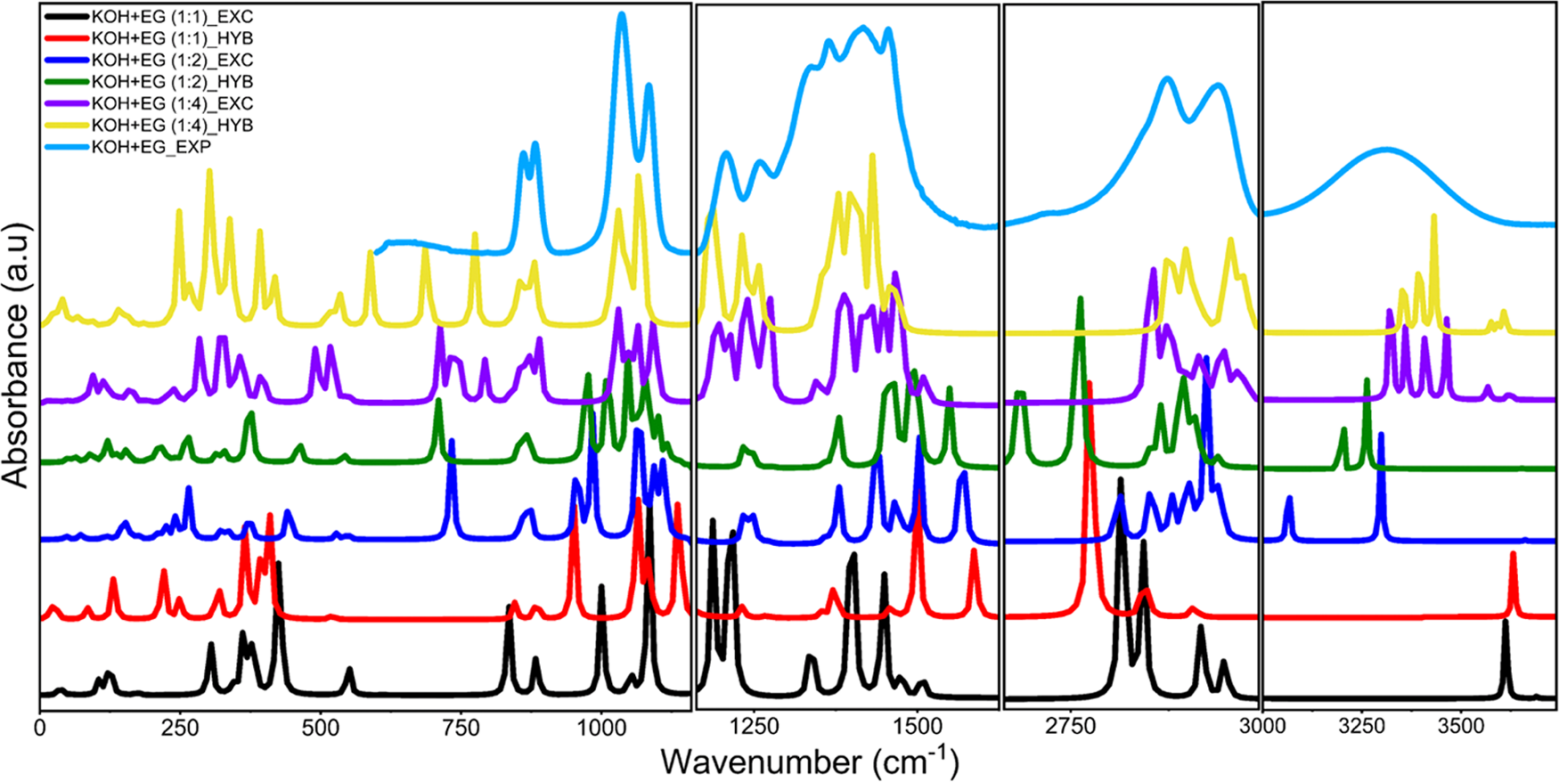
Comparison between calculated IR spectra for various KOH-EG ratios in explicit (EXC) and hybrid (HYB) models and experimentally (EXP) observed spectra for 1 M KOH in EG.

A comparison of calculated spectra for KOH, EG, and the 1:1 KOH-EG complex for both explicit and hybrid models with the experimental spectra is presented in Figure 7. A blue shift in OH stretching peaks is observed for the KOH-EG 1:1 ratio for both explicit and hybrid models in comparison to H-bonding stretching modes of EG. This supports the observation of blue shift in the OH stretching frequency region in the experimental spectra after adding KOH to EG. However, the blue shift is slightly more for the hybrid model suggesting that the type of interactions gleaned from the calculation depends on whether a continuum solvation model is employed. As before, the calculations do not exhibit the peak broadening due to H-bonding in the OH stretching frequency regions. Therefore, we carried out additional calculations increasing the number of EG molecules such that ratios of 1:2 and 1:4 were also probed. The computed vibrational spectra are presented in Figure 8 along with the experimentally observed spectra, and the calculated frequencies are given in Table S5 in the SI. As can be seen from this figure, the calculated spectra for the 1:4 KOH-EG system with a continuum solvation environment closely reproduce the experimental spectra. Hence, we focus on this system and perform further analyses.

#### Assessment of EG Polarization in EG-KOH Mixtures

**Figure 9:**
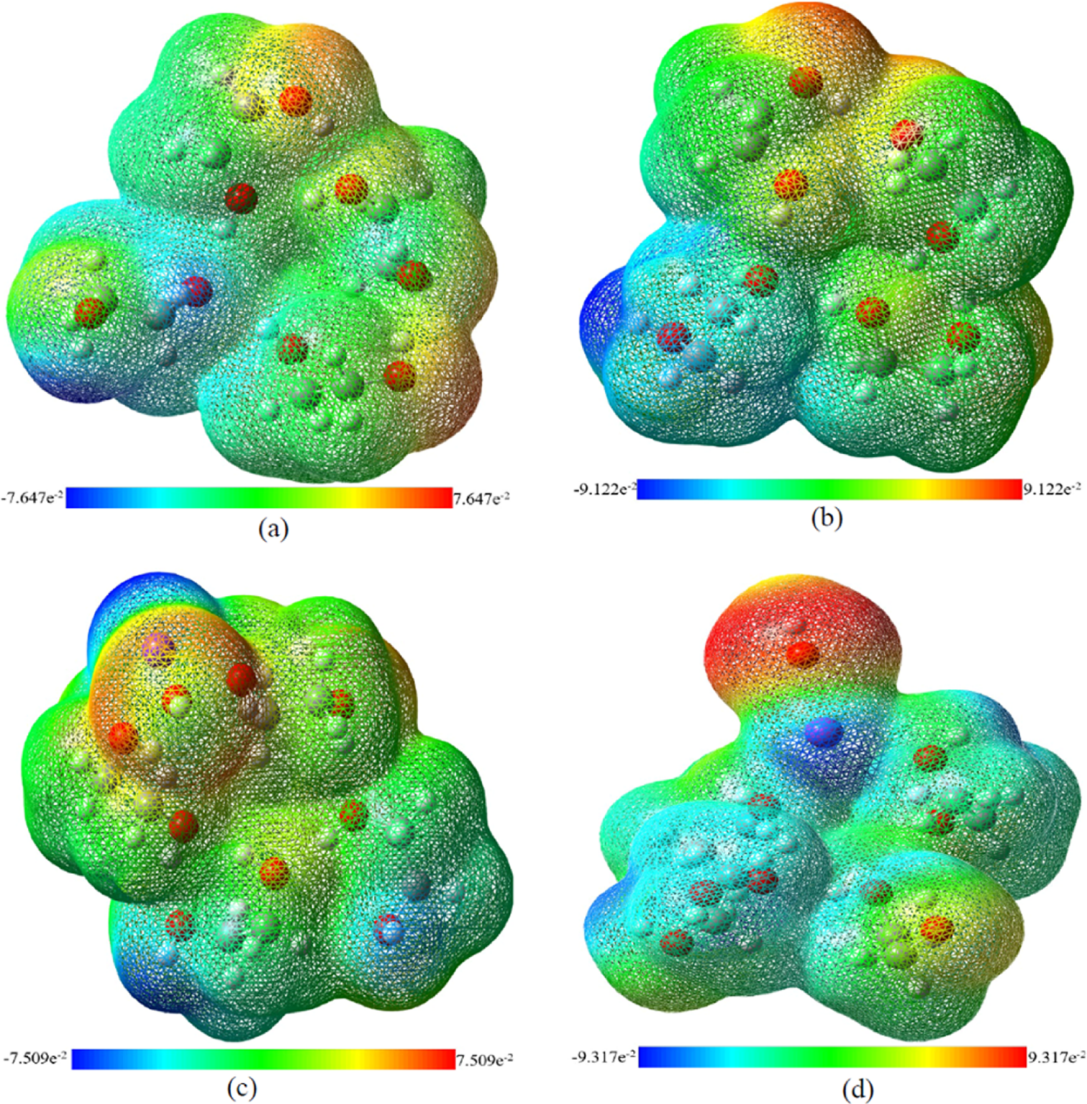
Comparison of MESP surfaces formed by mapping total density over the electrostatic potential of EG tetramer in (a) explicit and (b) hybrid models and KOH-EG complexes for a 1:4 ratio in (c) explicit and (d) hybrid models.

Polarization is an essential component of intermolecular interactions, and polarizable solvents enhance CO_2_ absorption by inducing dipole interactions, increasing solubility and absorption capacity. However, higher polarizability can increase viscosity, potentially slowing CO_2_ diffusion and absorption rates. Therefore, MESP analysis^41,42,91^ was performed to analyze the changes in the polarization for EG upon the addition of KOH. The MESP plots for explicit and hybrid models for EG monomer are displayed in Figure S1a,b in the SI. These plots show a region, depicted in red and surrounded by another yellow region, indicative of weak negative potential localized over the oxygen atoms of the hydroxyl groups. The highest positive potential, represented in blue, is found on the hydrogen atom of the hydroxyl groups. As expected, increasing the number of EG molecules results in a corresponding increase in the regions where negative and positive potentials are localized, as shown in Figure S2a,b in the SI and Figure 9a,b. The presence of continuum solvation in the hybrid model does not seem to affect the electropositive and electronegative regions observed in the explicit model.

To provide a quantitative measure of the degree to which atoms, ions, or molecules are polarized is typically explained by their chemical hardness η.^62,63^ It provides resistance against the change in electron distribution, indicating the extent of electron cloud distortion in an electric field. The calculated values of η are provided in Table 3. The chemical hardness decreases with an increase in the number of EG molecules, indicating that the system becomes more polarizable. The polarity is higher for hybrid models than for explicit models due to solvent effects. In the presence of KOH, the electropositive area is found to delocalize and distribute over the complex as shown in Figure S1c,d in the SI for a 1:1, in Figure S2c,d in the SI for a 1:2, and in Figure 9c,d for 1:4 KOH-EG ratio. A strong positive potential is mainly confined to K^+^ ions, and a weak positive potential is found for one or more hydrogen atoms of the hydroxyl group. On the other hand, OH^–^ is the primary site for a strong negative potential, while only a mild electronegative potential is observed on the oxygen atoms of EG. It can be observed that the addition of KOH leads to elevated electronegativity and electropositivity as suggested by the range presented in Figure 9. The decrease in η values after adding KOH indicates an enhancement in the polarity and polarizability of the system, which supports the observation of the FT-IR spectra of the increase in polarizability as the reason behind the formation of improper H-bonds and peak broadening.

**Table 3:** Comparison of Chemical Hardness η (eV) of EG Oligomers and KOH-EG Complexes for Both Explicit (EXC) and Hybrid (HYB) Models

EG						KOH-EG complexes					
monomer		dimer		tetramer		1:1 ratio		1:2 ratio		1:4 ratio	
EXC	HYB	EXC	HYB	EXC	HYB	EXC	HYB	EXC	HYB	EXC	HYB
7.01	7.37	6.40	7.09	5.66	6.69	3.74	5.93	6.09	6.50	3.91	5.24

#### Assessment of Proper and Improper H-Bonding

To differentiate between the proper and improper H-bond interactions as observed by vibrational spectra analysis natural bond orbital (NBO) analysis was performed.^92^ In a proper H-bond, charge transfer occurs from lone pair (LP) electrons of the hydrogen bond acceptor atom to the antibonding orbital (AB) of the O–H bond, resulting in elongation of the H-bond. In the improper H-bond, O–H bond contraction occurs due to the charge transfer to the remote parts of the donor molecule.^92^

The charge transfer analysis performed by NBO calculations is presented in Table S6a for EG (monomer, dimer), in Table S6b for KOH+EG 1:1 and 1:2 ratios, and in Table S6c for EG tetramer, and KOH-EG 1:4 ratio in the SI for the explicit and hybrid models. The results of the gas-phase EG dimer show that all of the H-bonds in the dimer arise due to intermolecular interactions and there are no intramolecular H-bonds, which is consistent with earlier studies.^86^ The essential interactions of EG oligomers leading to high stabilization energies are from the lone pair of the oxygen atom of one EG molecule to the antibonding orbitals of the hydroxyl group of the neighboring EG molecule. This indicates the formation of proper H-bonds.^92^ However, for the EG tetramer, some charge transfers are from lone pair to neighboring parts of the donor atom for the C–H–O type H-bonds, suggesting that as the number of molecules of EG increases, different types of H-bonds appear. The varied nature of H-bonding was reflected in the breadth of the IR spectra of EG. Additional confirmation of the H-bonding interaction can be gleaned from high *E*^(2)^ values^93^ between EG and KOH. In the case of the KOH-EG complex, mostly the charge transfers are from the LP of OH^–^ ions to the antibonding orbital of the neighboring hydroxyl group of the EG molecule. However, some of the charge transfers occur from the LP on the oxygen of the hydroxyl ion to the antibonding orbitals of the C–H bond in EG. This is an indication of improper H-bond formation leading to smaller H-bond lengths in comparison to proper H-bonds between hydrogen and hydroxyl groups leading to a blue shift in the FT-IR spectra.

#### Assessment of Interactions beyond H-Bonding

To confirm and analyze other intermolecular interactions apart from H-bonding that also influence CO_2_ absorption, QTAIM analysis was performed. It helps to analyze the electronic structure (topological features of electron density) and bonding characteristics (nature of chemical and molecular interactions) of molecules based on electron density distribution.^94^ It represents all of the possible interactions in a system. A thorough analysis of the values of topological descriptors at the bond critical point (BCP), namely, the electron density (ρ_BCP_), the Laplacian of the electron density (∇^2^ρ_BCP_), the Lagrangian kinetic energy (*G*_BCP_), the potential electron density (*V*_BCP_), the energy density (*H*_BCP_), and the binding energy (BE_BCP_), was conducted to characterize H-bonds at the BCPs.^94−96^ The analysis is valuable for understanding chemical bonds, especially H-bonds, which can be identified as a value of ρ_BCP_ between 0.002 au and 0.040 au and ∇^2^ρ_BCP_ between 0.02 au and 0.15 au. In conjunction with the analysis, the topology maps are employed to exhibit the existence and positions of BCPs, indicating the formation of intermolecular interactions.^96^

**Figure 10:**
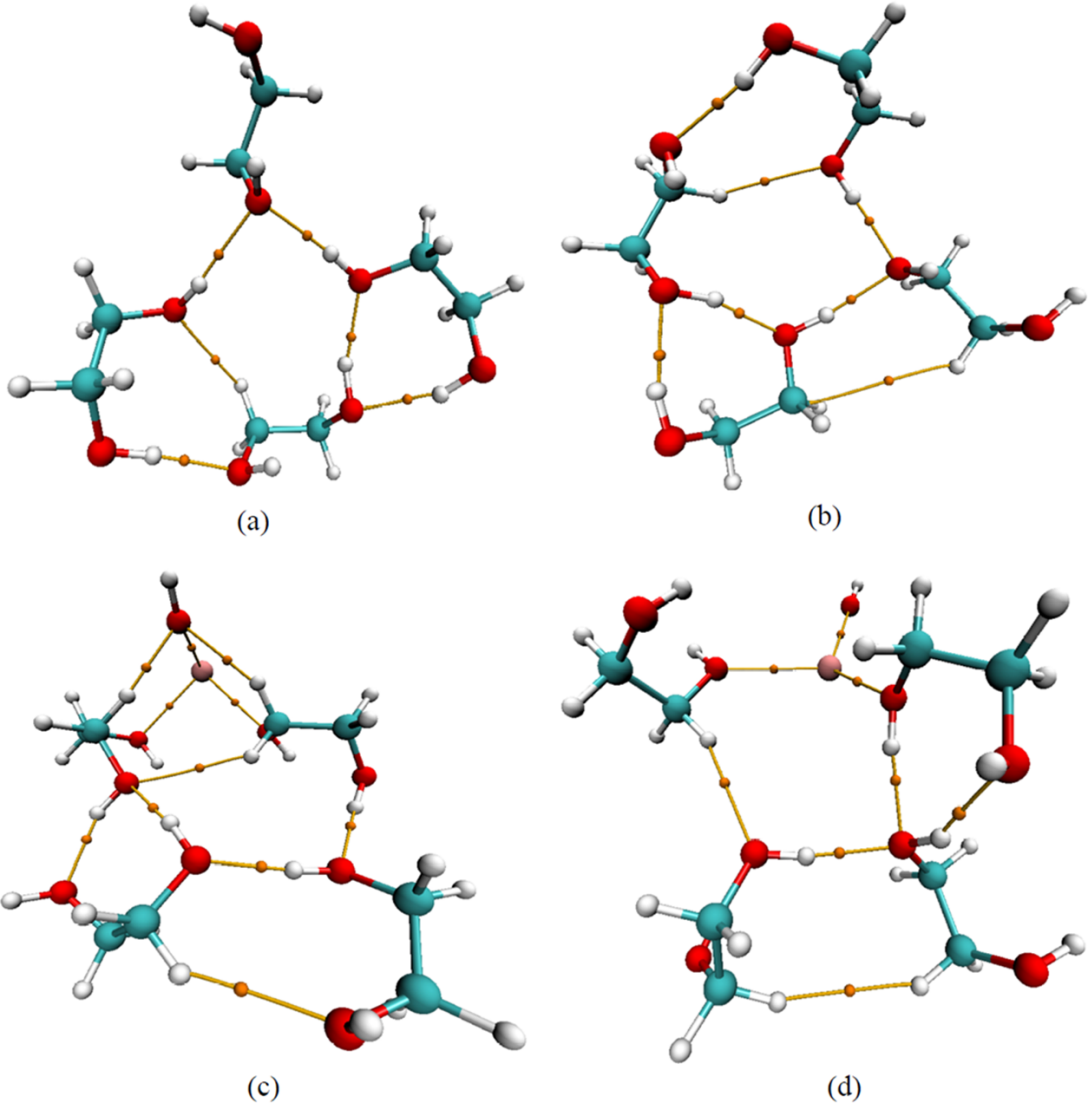
Comparison of QTAIM molecular graphs of EG tetramer for (a) explicit and (b) hybrid model and KOH-EG 1:4 ratio for (c) explicit and (d) hybrid models. Blue, red, white, and mauve colors represent C, O, H, and K atoms, respectively. The orange line and point represent intermolecular interactions and BCP, respectively.

Figure 10a,b shows the topology maps showing the various interactions for EG tetramer, for both explicit and hybrid models, respectively. Refer to Figure S3a,b for the corresponding figures for the EG dimer. In the case of explicit model (Figure 10a), all BCPs refer to the H-bonding interactions, both C–H···O H-bonds and relatively strong O–H···O bonds,^85,90^ aligning well with earlier experimental studies^89^ and current geometry studies and vibrational analysis. The strength of the H-bonds varies from −1.3 to −7.2 kcal/mol (Table S7a). The absence of BCP within EG molecules is consistent with the results of geometry optimization and NBO analysis that there are no intramolecular interactions.^85^ In the case of EG tetramer placed in a continuum solvation model, an additional van der Waals-type interaction appears between two EG molecules, which is elaborated in the subsequent section (Table S7a) on the noncovalent interaction present in these systems. The H-bonds are strengthened in comparison to those in the explicit environment such that the strength varies from −1.0 to −8.0 kcal/mol.

The topology maps for the KOH-EG system for a 1:4 ratio are presented in Figure 10c,d for the explicit and hybrid models, respectively. Additional topology maps for KOH-EG ratios of 1:1 and 1:2 are provided in Figures S4a,b and S3c,d, respectively. It can be seen in Figure 10c that new BCPs, involving interactions between EG and K^+^ and EG and OH^–^ appear. These include improper H-bonding interaction between the oxygen of OH^–^ and C–H of two different EG molecules, while K^+^ interacts with an oxygen atom in EG. The H-bonds established between EG molecules are on the order of −5.5 kcal/mol, except for one H-bond. On the other hand, the H-bonds with OH^–^ are significantly weaker with an average strength of ca. −3.1 kcal/mol (refer Table S7b). Since proper H-bonds are stable, they have higher binding energy as compared to improper H-bonds which are less stable. The weakening of H-bonds of EG in the presence of KOH is the primary reason for the blue shift observed in the experimentally measured spectra. This weakening of H-bonds in the case of KOH-EG 1:4 ratio complexes indicates a possible decrease in viscosity which could facilitate faster diffusion of CO_2_ once absorbed.

The values reported in Table S7a for EG oligomers and Table S7b for all KOH-EG complexes confirm the existence of H-bonds in both the EG oligomers and KOH-EG clusters. Higher electron density values, ρ(*r*), indicate stronger interactions. Adding KOH, reduces H-bond electron density, demonstrating a weakening of H-bond interactions for proper H-bonds. An increase in H-bond electron density indicates a stronger H-bond interaction supporting earlier analysis of the presence of improper H-bonds. ∇^2^ρ_BCP_ > 0 for all reported bonds indicates electron segregation and the existence of ionic bonds, H-bonds, and van der Waals (VDW) interactions. Rozas et al. classified weak H-bonds as ∇^2^ρ_BCP_ > 0 and *G*_BCP_ + *V*_BCP_ > 0; medium H-bonds as ∇^2^ρ_BCP_ > 0 and *G*_BCP_ + *V*_BCP_ < 0; strong H-bonds as ∇^2^ρ_BCP_ < 0 and *G*_BCP_ + *V*_BCP_ < 0. The bonds having ∇^2^ρ_BCP_ > 0 and *H*_BCP_ < 0, such as the bond O1H39 for the EG tetramer in both explicit and hybrid models, the bonds in the KOH-EG 1:1 ratio for the hybrid model, the bonds in the KOH-EG 1:2 ratio for both explicit and hybrid model are classified as strong H-bonds and are covalent dominant. The remaining H-bonds are categorized as weak H-bonds, which are electrostatically dominant as ∇^2^ρ_BCP_ > 0 and *H*_BCP_ > 0. The BE_BCP_ values are also in alignment with those of the above analysis. This indicates that for a higher EG ratio, the H-bonds are medium or weak, and involve proper H-bonds or other intermolecular interactions. This also confirms the presence of different types of H-bonds (proper and improper) as observed by FT-IR spectra. The topology maps and the topological descriptors confirm the presence of other noncovalent interactions, apart from H-bonding, as they have additional BCPs not categorized as H-bonds by geometry optimization results. To visualize the differences in the interaction and to support the analysis of the presence of other noncovalent interactions, RDG-NCI is performed.

RDG-NCI investigation^97−99^ is an extension to QTAIM, which aids in visualizing and deciphering the differences in intermolecular interactions such as H-bonding, van der Waals forces, steric interactions etc.^46,100,101^ The RDG-NCI analysis involves determining the electronic density and the eigenvalue (λ_2_) of the electron density Hessian matrix.^101^ Based on the product of the electron density and the sign of λ_2_, various interactions are identified. For example, ρ > 0 and *sign*(λ_2_)ρ < 0 is suggestive of a H-bonding interaction. Furthermore, the magnitude of *sign*(λ_2_)ρ < 0 can be used to assess the strength of the H-bond, with more negative values indicating stronger H-bonding interactions.^101^ The presence of a van der Waals interaction is detected when *sign*(λ_2_)ρ ∼ 0, while *sign*(λ_2_)ρ > 0 points to the existence of strong repulsion.

**Figure 11:**
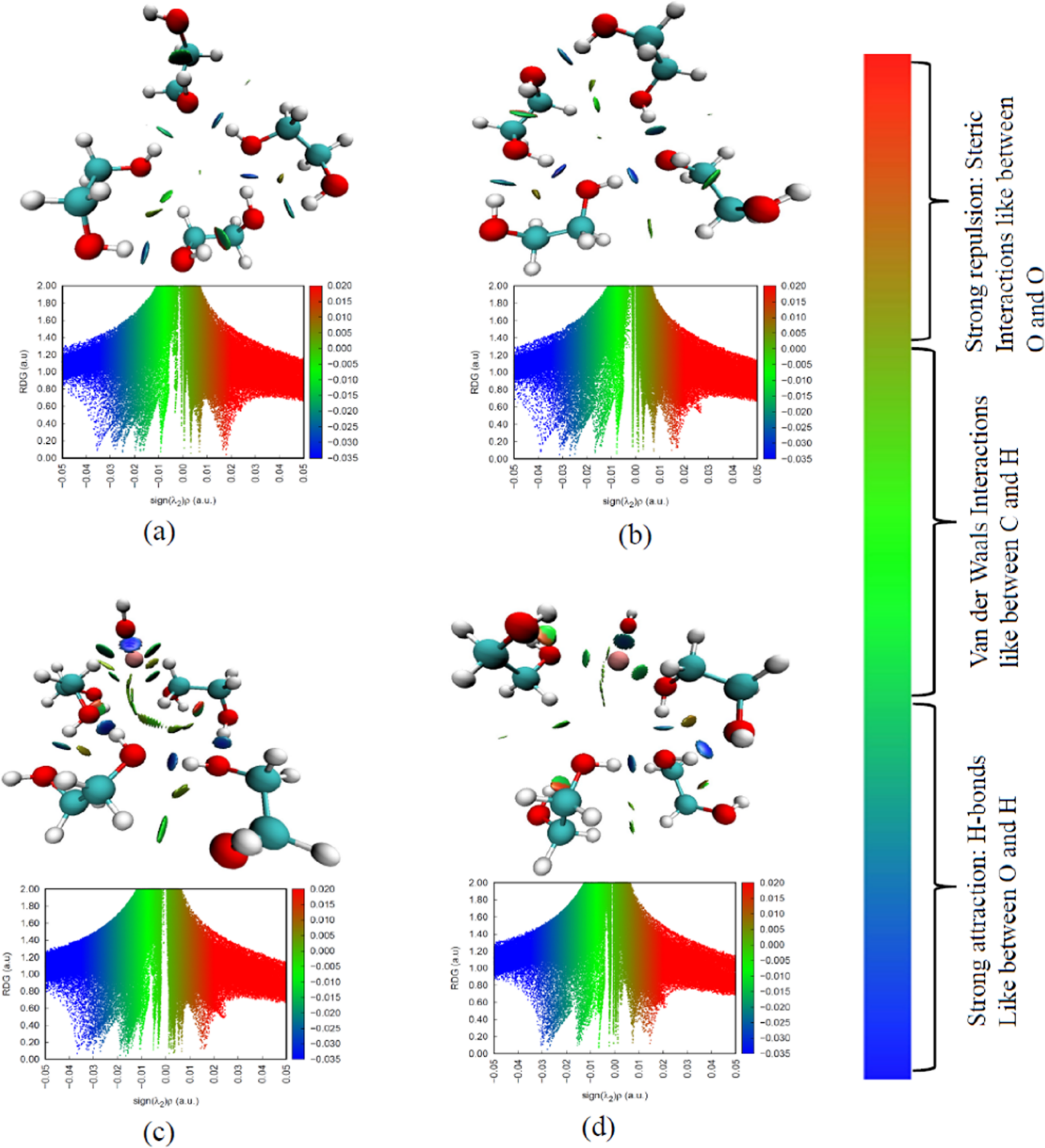
Comparison of NCI and RDG plots of EG tetramer for (a) explicit and (b) hybrid models and KOH-EG 1:4 ratio for (c) explicit and (d) hybrid models. Blue, red, white, and mauve colors represent C, O, H, and K atoms, respectively.

The RDG-NCI plots for the EG tetramer are depicted in Figure 11a,b, while those for the dimer are given in Figure S5a,b in the SI. Consistent with the QTAIM results, the tetramer shows five strong H-bonds as indicated by blue circular disks and a weak H-bond (represented by a green disk) for the explicit model. Based on the more negative values of *sign*(λ_2_)ρ, it can be concluded that H-bonding is more pronounced in the hybrid model. All of the NCI-RDG plots confirm the presence of some van der Waals interactions among EG molecules, as suggested by *sign*(λ_2_)ρ ∼ 0 in the RDG plots. The change in the interactions as the number of EG molecules explicitly modeled is varied can be observed in RDG-NCI plots portrayed in Figure 11c,d for a 1:4 ratio of KOH-EG. Similar plots are provided in Figure S4c,d for 1:1 and Figure S5c,d for 1:2 ratios. For KOH-EG 1:4 ratio, the maps show four new interactions, composed of two van der Waals interactions between K^+^ and hydroxyl groups of EG represented by green circular disks and two weak H-bonds between OH^–^ ion and H of EG indicated by green disks with a slight blue color in the middle. Blue disks represent the remaining H-bonds between EG molecules. Some of the H-bonds present in EG tetramer are weakened and changed to van der Waals interaction, implying disruption in H-bonding network. A dark blue circular disk with a red outline represents a strong interaction between K^+^ and OH^–^ ions due to ion-dipole and Coulombic interactions. Some of the red areas represent steric interactions. The H-bonding visualized and depicted by RDG plots agrees well with the optimized geometrical parameters and QTAIM results. It shows the visual location and differences among the van der Waals interactions, steric interactions, and H-bonds predicted by QTAIM. van der Waals and other ionic interactions also contribute to the stability and solubility of CO_2_ in the solvent, maintaining the solvent structure while accommodating CO_2_ molecules. This information about these interactions is also essential for CO_2_ absorption and designing the DES system for desired CO_2_ solubility as they affect viscosity.^21^

## Conclusions

The study focuses on a comprehensive understanding of the molecular interactions within EG oligomers and KOH-EG complexes utilizing vibrational spectroscopy in conjunction with quantum chemical calculations and on how these interactions could affect CO_2_ absorption. The FT-IR spectra obtained from experiments show that there is a blue shift in the OH stretching frequency region and peak broadening as KOH is added to EG. The magnitude of the shift and the peak broadening is proportional to the molar ratio of KOH in EG. The blue shift and peak broadening are possibly due to the formation of improper H-bonds and the weakening of H-bonds in EG. In order to explain the experimental spectra, quantum mechanical calculations were carried out for various KOH-EG ratios. A critical aspect of our investigation was to understand the effect of explicit and hybrid models in reproducing the vibrational spectra. Analysis of optimized geometries and interaction energy confirmed the presence of interactions between KOH and EG. Our computational analysis revealed the presence of noncovalent interactions such as H-bonding (proper and improper H-bonds) among EG molecules and between OH^–^ and EG. Furthermore, our investigation of the KOH-EG system through MESP analysis revealed an enhancement in polarizability after adding KOH to EG. Charge transfer analysis confirmed the presence of improper H-bonds. QTAIM analysis showed the presence of other noncovalent interactions apart from H-bonding, such as van der Waals and steric interactions. RDG-NCI analysis supported the QTAIM results and provided deeper insight into the nature and strength of the interactions. The total interaction between KOH and EG is a subtle balance between Coulomb forces, H-bonds, and van der Waals interactions. These all interactions are influenced by the molar ratio of KOH and EG and affect viscosity, thereby impacting CO_2_ absorption efficiency. The results provided information about the optimal ratio of DES components for better CO_2_ absorption. The information provided herein could serve as a basis for further studies on different derivatives of EG, other cosolvents and alkali hydroxides for CO_2_ absorption in direct air capture processes. In addition, salts other than KOH may also be used. Studies on some of these systems are currently underway in our research group.

## Data Availability

Gaussian input files and sample analysis files can be downloaded from https://github.com/ShahResearchGroup/EG_KOH_QM_JPCB_2024.git.

## Supporting Information Available

Electrostatic maps, RDG and NCI plots, coordinates of optimized structures, comparison of IR modes for various ratios and models, data on second-order perturbation analysis, and data for various quantities derived from electron density in RDG analysis (PDF)
