# Supplementary material for: Role of Intermolecular Interactions in Deep Eutectic Solvents for CO2 Capture: Vibrational Spectroscopy and Quantum Chemical Studies
Source: J Phys Chem B. 2024 Oct 9;128(41):10214–29. doi: 10.1021/acs.jpcb.4c04509 (PMC11492266; doi:10.1021/acs.jpcb.4c04509)
Supplement: Supplementary file 1 — jp4c04509_si_001.pdf [file jp4c04509_si_001.pdf]

Supporting Information to the article "Role of Intermolecular Interactions in Deep Eutectic Solvents for CO_2 Capture: Vibrational Spectroscopy and Quantum Chemical Studies"

Rashmi Mishra, [†] Rajan Bhawnani, [‡] Rohan Sartape, [‡] Rohit Chauhan, [‡] Amey S. Thorat, [†] Meenesh R. Singh,[‡] Jindal K. Shah[*,†]

[†]School of Chemical Engineering, Oklahoma State University, 420 Engineering North,

Stillwater, Oklahoma, 74078, USA

[‡]Department of Chemical Engineering, University of Illinois at Chicago, 929 W. Taylor

St., Chicago, Illinois, 60607, USA

E-mail: jindal.shah@okstate.edu

Figure S1 A comparison of MESP surfaces of EG monomer (a) explicit and (b) hybrid model and a mixture of KOH and EG (1:1 ratio) explicit (c) and (d) hybrid model.

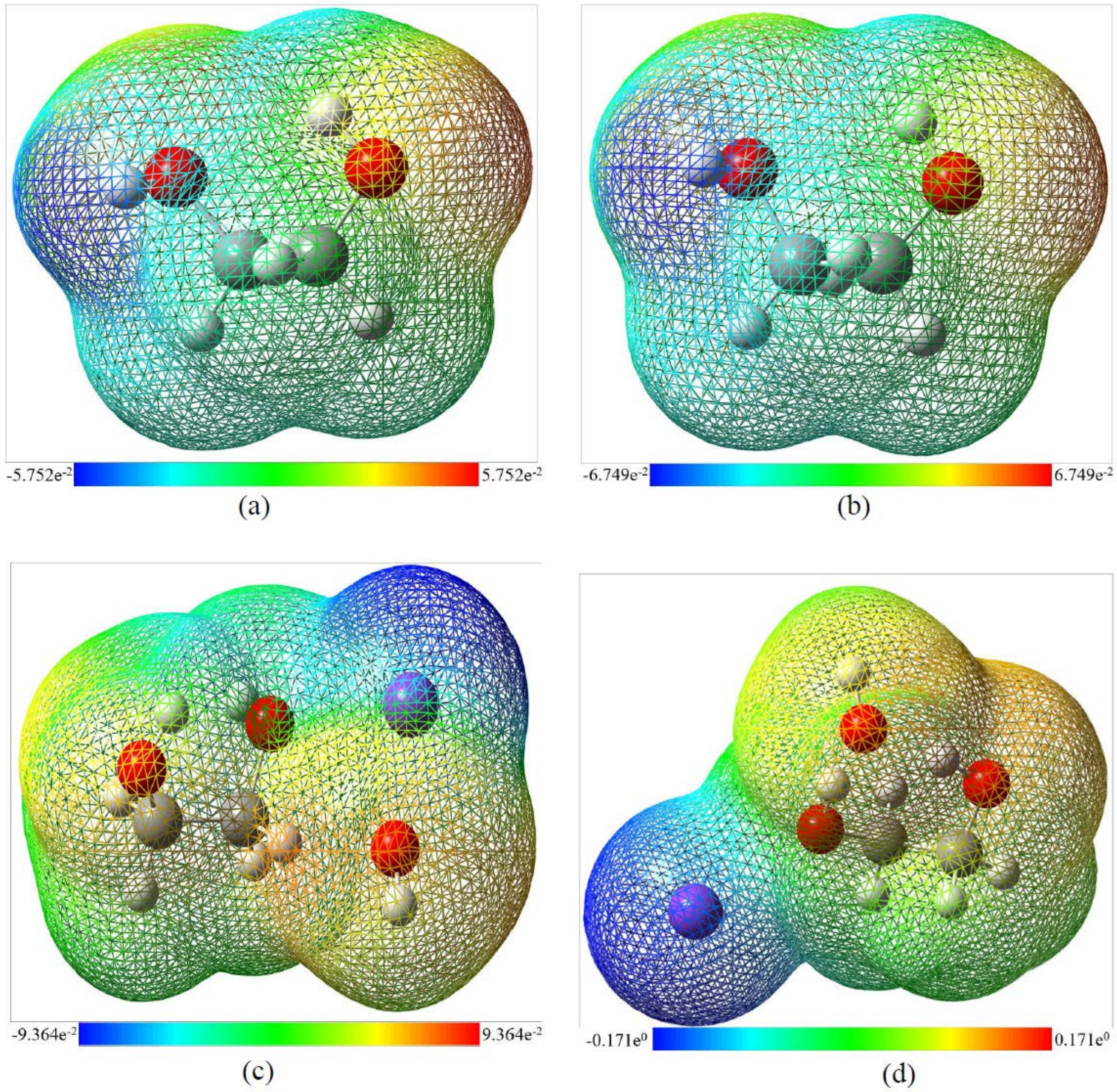

Figure S2 A comparison of MESP surfaces of EG dimer (a) explicit and (b) hybrid model and a mixture of KOH and EG (1:2 ratio) explicit (c) and (d) hybrid model.

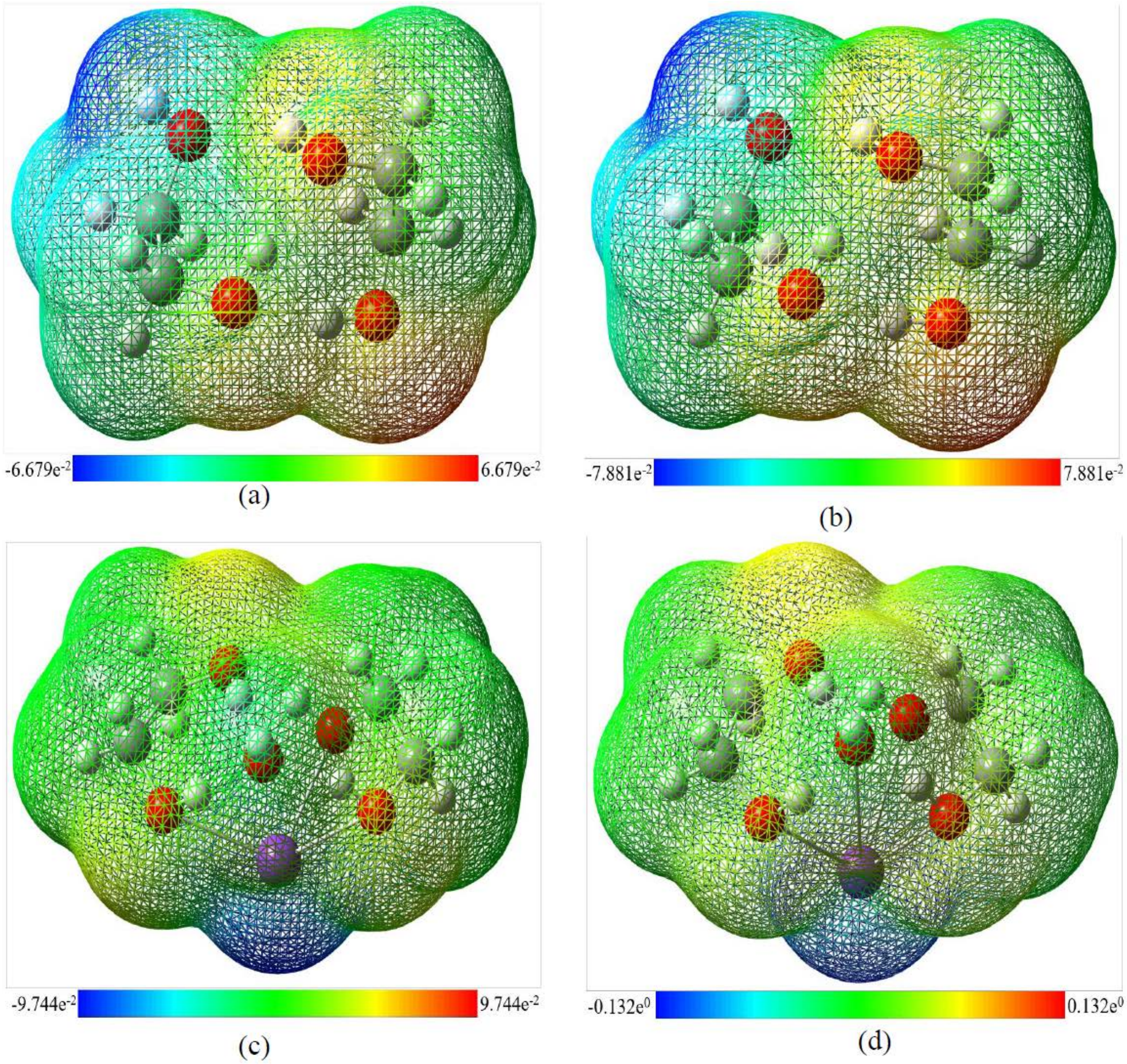

Figure S3 A comparison o[illegible]olecular graphs of EG dimer for (a) explicit and (b) hybrid model and KOH-EG (1:2) (c) explicit and (d) hybrid model.

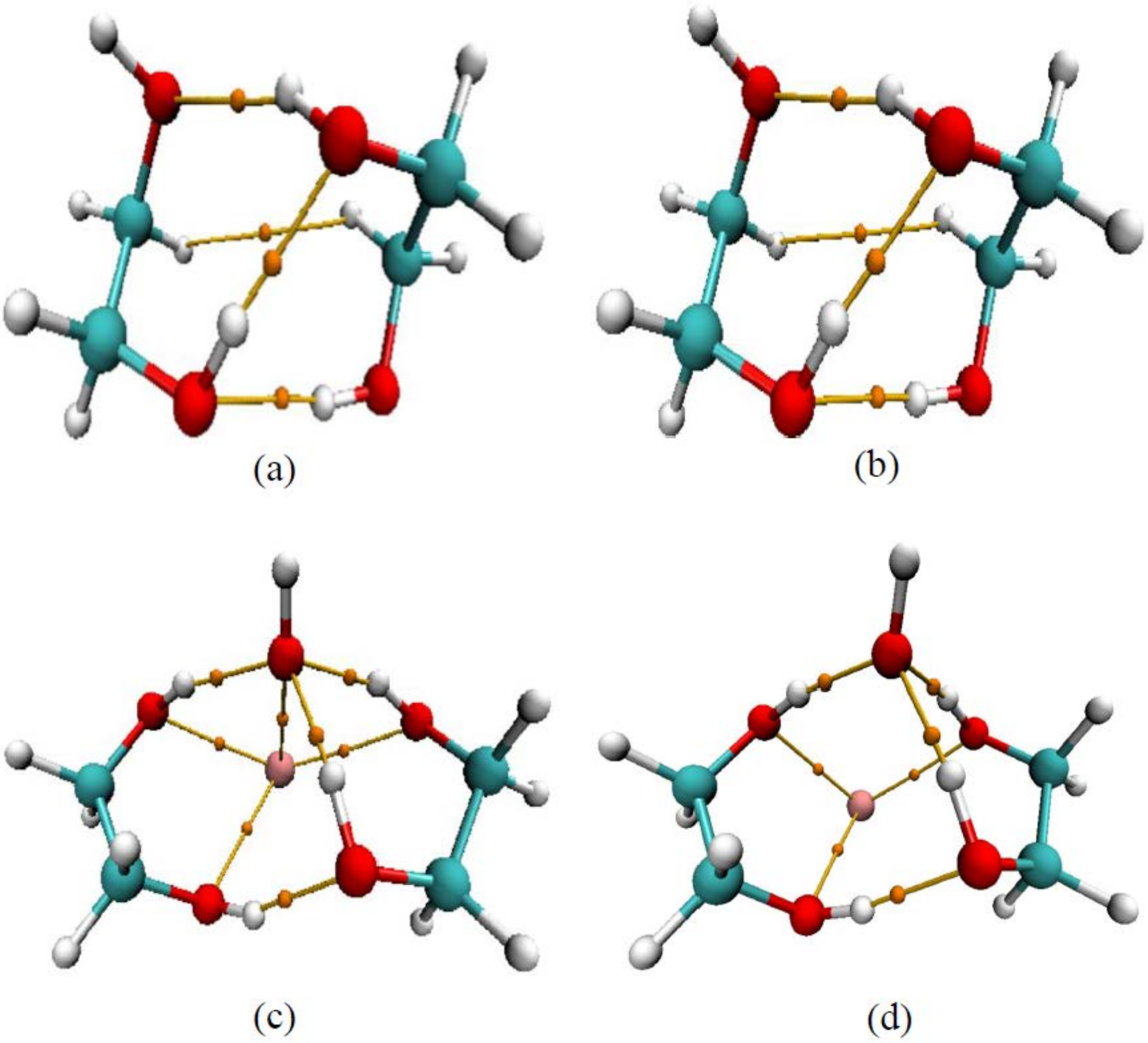

Figure S4 A comparison of KOH-EG (1:1) molecular graphs (a) explicit and (b) hybrid model and NCI and RDG plots (c) explicit and (d) hybrid model.

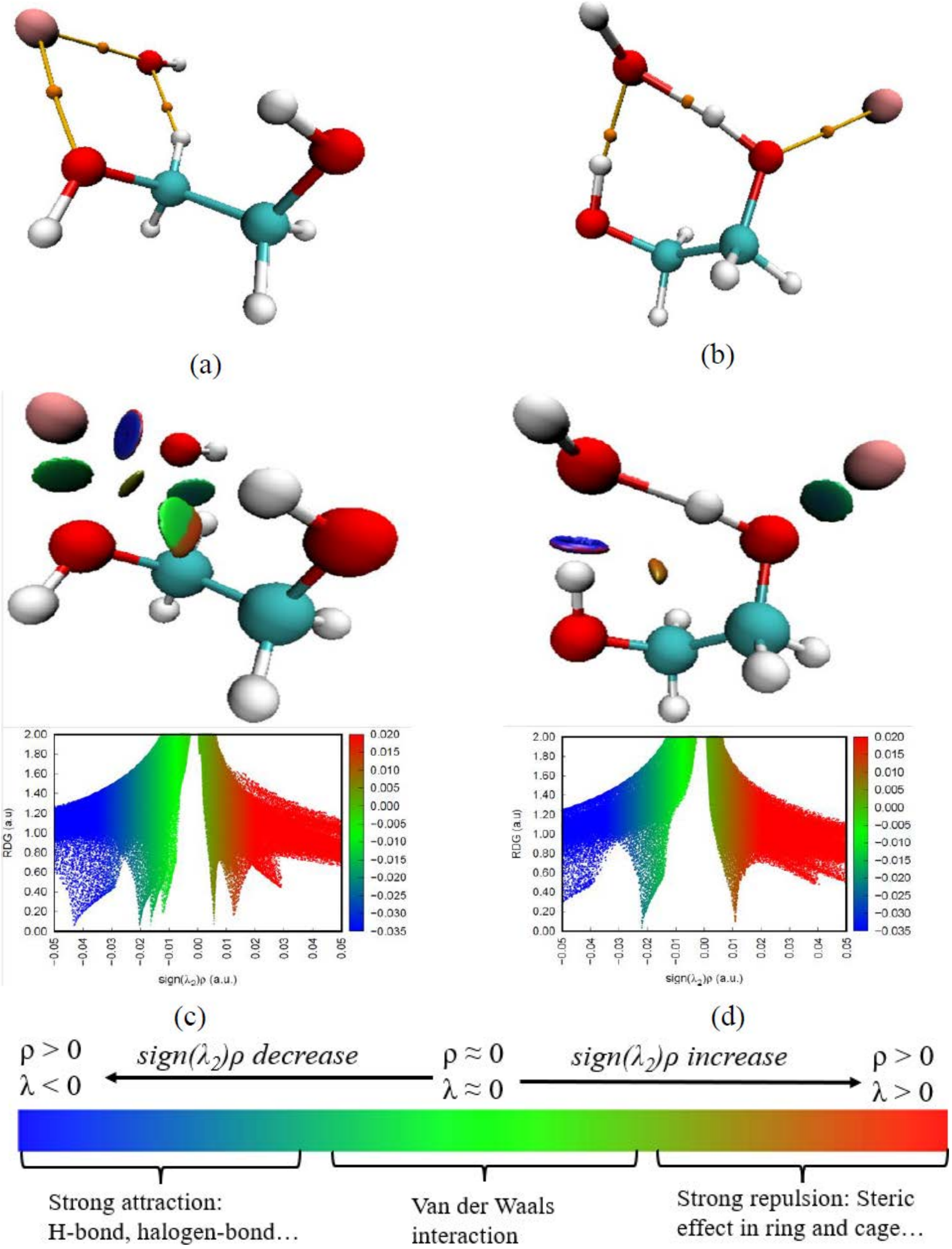

Figure S5 A comparison of NCI and RDG plots of EG dimer for (a) explicit and (b) hybrid model and KOH-EG 1:2 ratio (c) explicit and (d) hybrid models.

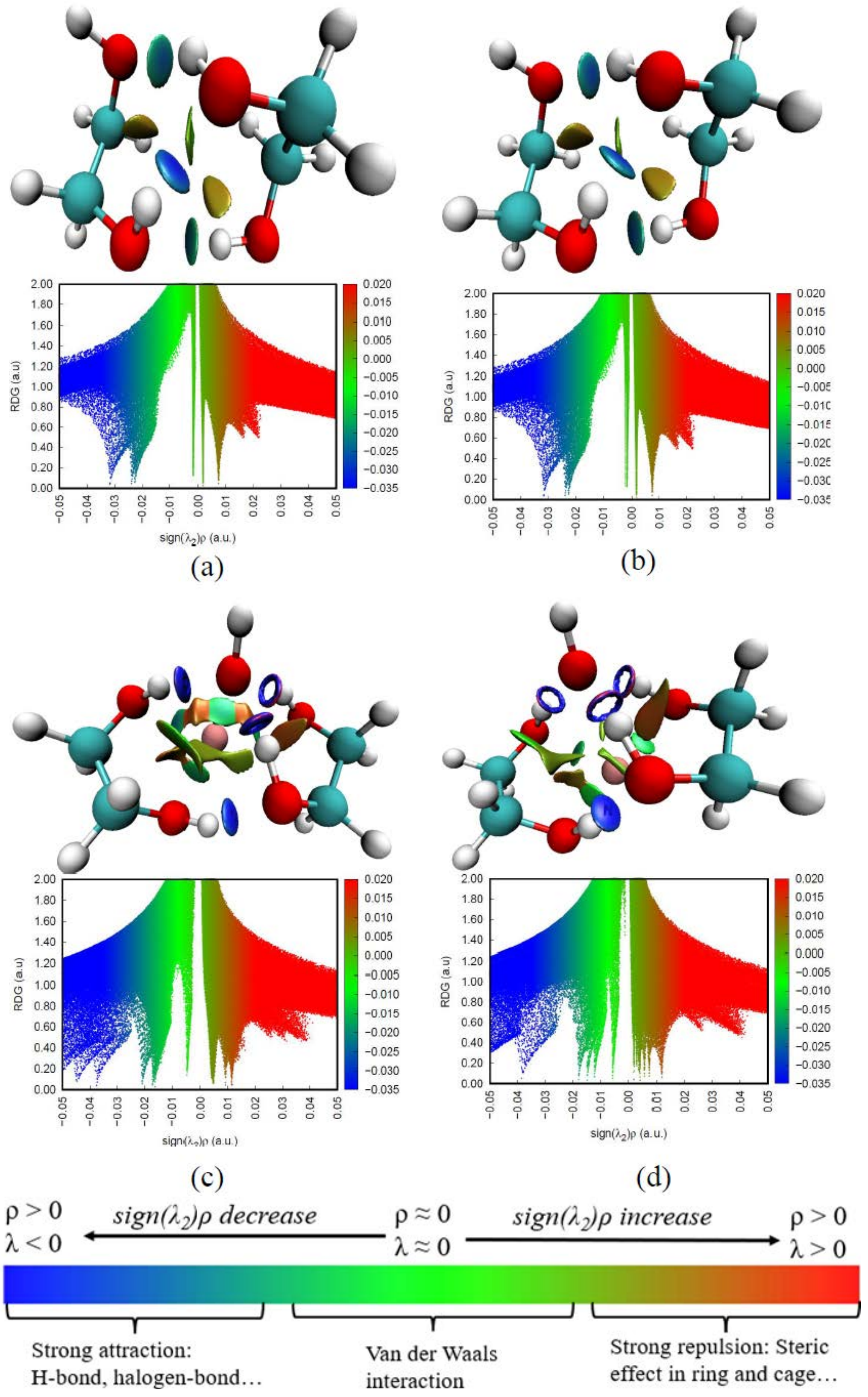

Table S1 (a) A comparison of the measured center frequencies (cm^{-1}) of the prominent IR peaks observed in the FT-IR spectra of EG and KOH-EG complexes at various molar ratios.

Molar ratios	ν(OH)	ν(CH)		ν(CC)	ν(CO)	$\beta(CH_2)$	ν(CC)+ν(CO)
EG	3300.520	2939.866	2875.269	1084.212	1033.854	881.949	861.223
EG + 0.25M KOH	3307.667	2939.700	2874.554	1084.212	1034.633	881.949	861.223
EG + 0.50M KOH	3311.241	2938.878	2874.554	1084.212	1035.717	881.949	861.223
EG + 0.75M KOH	3314.100	2938.786	2873.125	1084.212	1036.326	881.949	861.223
EG + 1.00M KOH	3314.100	2938.163	2873.125	1084.212	1035.612	881.949	861.223

Types of vibration: υ, stretching; β, bending.

Table S1 (b) A comparison of the full width at half maximum (FWHM) in radians (rad) for the prominent peaks observed in the FT-IR spectra of EG and KOH-EG complexes at various molar ratios.

Molar ratios	ν(OH)	ν(CH)		ν(CC)	ν(CO)	$\beta(CH_2)$	ν(CC)+ν(CO)
EG	4.683	0.656	2.24	0.456	0.648	0.396	0.381
EG + 0.25M KOH	4.662	0.637	2.253	0.453	0.647	0.34	0.302
EG + 0.50M KOH	4.759	0.624	2.368	0.443	0.652	0.337	0.309
EG + 0.75M KOH	4.809	0.609	2.561	0.44	0.657	0.335	0.315
EG + 1.00M KOH	4.878	0.604	2.678	0.437	0.674	0.66	0.004

Types of vibration: υ, stretching; β, bending.

Table S2 PDB files for EG (dimer and tetramer) for both explicit and hybrid model.

S2 (a) EG - Dimer explicit model

```
HETATM   1  O     0   -1.583   1.411  -0.509      O
HETATM   2  O     0   -0.816  -1.188   0.835      O
HETATM   3  C     0   -2.124   0.110  -0.802      C
HETATM   4  C     0   -2.117  -0.829   0.399      C
HETATM   5  H     0   -3.145   0.207  -1.191      H
HETATM   6  H     0   -1.495  -0.298  -1.595      H
HETATM   7  H     0   -2.621  -1.758   0.116      H
HETATM   8  H     0   -2.692  -0.382   1.224      H
HETATM   9  H     0   -2.225   1.910   0.007      H
HETATM  10  H     0   -0.337  -0.397   1.148      H
HETATM  11  O     0    0.824   1.050   0.965      O
HETATM  12  O     0    1.794  -1.463  -0.463      O
HETATM  13  C     0    2.117   0.837   0.383      C
HETATM  14  C     0    2.100  -0.123  -0.803      C
HETATM  15  H     0    2.546   1.797   0.066      H
HETATM  16  H     0    2.739   0.427   1.180      H
HETATM  17  H     0    3.102  -0.139  -1.244      H
HETATM  18  H     0    1.413   0.261  -1.574      H
HETATM  19  H     0    0.226   1.442   0.308      H
HETATM  20  H     0    0.883  -1.516  -0.133      H
END
```

S2 (b) EG - Dimer hybrid model

```
HETATM   1 O    0   -1.540  1.424 -0.474    O
HETATM   2 O    0   -0.836 -1.190  0.833    O
HETATM   3 C    0   -2.112  0.144 -0.807    C
HETATM   4 C    0   -2.138 -0.820  0.370    C
HETATM   5 H    0   -3.128  0.275 -1.192    H
HETATM   6 H    0   -1.490 -0.258 -1.609    H
HETATM   7 H    0   -2.631 -1.743  0.055    H
HETATM   8 H    0   -2.722 -0.391  1.194    H
HETATM   9 H    0   -2.178  1.925  0.049    H
HETATM  10 H    0   -0.366 -0.394  1.147    H
HETATM  11 O    0    0.856  1.045  0.971    O
HETATM  12 O    0    1.746 -1.483 -0.421    O
HETATM  13 C    0    2.132  0.836  0.347    C
HETATM  14 C    0    2.090 -0.155 -0.810    C
HETATM  15 H    0    2.536  1.789 -0.015    H
HETATM  16 H    0    2.791  0.460  1.133    H
HETATM  17 H    0    3.089 -0.207 -1.252    H
HETATM  18 H    0    1.406  0.210 -1.588    H
HETATM  19 H    0    0.227  1.423  0.330    H
HETATM  20 H    0    0.819 -1.494 -0.126    H
END
```

S2 (c) EG - Tetramer explicit model

```
HETATM   1 O    0   -1.126 -1.617  0.237    O
HETATM   2 O    0   -0.325 -4.217 -1.103    O
HETATM   3 C    0   -2.012 -2.589 -0.342    C
HETATM   4 C    0   -1.408 -3.980 -0.224    C
HETATM   5 H    0   -2.974 -2.571  0.188    H
HETATM   6 H    0   -2.185 -2.361 -1.400    H
HETATM   7 H    0   -2.184 -4.707 -0.484    H
HETATM   8 H    0   -1.124 -4.159  0.823    H
HETATM   9 H    0   -1.419 -0.720  0.005    H
HETATM  10 H    0    0.457 -3.740 -0.780    H
HETATM  11 O    0   -4.352  2.226 -0.660    O
HETATM  12 O    0   -1.846  1.103 -0.203    O
HETATM  13 C    0   -4.063  1.650  0.620    C
HETATM  14 C    0   -2.568  1.698  0.890    C
HETATM  15 H    0   -4.613  2.151  1.424    H
HETATM  16 H    0   -4.404  0.615  0.563    H
HETATM  17 H    0   -2.308  1.134  1.788    H
HETATM  18 H    0   -2.228  2.732  1.028    H
HETATM  19 H    0   -4.411  3.183 -0.576    H
HETATM  20 H    0   -2.284  1.395 -1.016    H
HETATM  21 O    0    0.814  2.060  0.677    O
HETATM  22 O    0    3.482  2.516 -0.754    O
HETATM  23 C    0    1.299  3.272  0.096    C
HETATM  24 C    0    2.225  3.056 -1.098    C
HETATM  25 H    0    0.462  3.918 -0.210    H
HETATM  26 H    0    1.854  3.787  0.884    H
HETATM  27 H    0    2.419  4.031 -1.559    H
```

```
HETATM   28  H          0       1.711   2.436  -1.851                   H
HETATM   29  H          0       0.090   1.716   0.135                   H
HETATM   30  H          0       3.397   1.568  -0.547                   H
HETATM   31  O          0       1.508  -2.312  -0.007                   O
HETATM   32  O          0       3.386  -0.281  -0.222                   O
HETATM   33  C          0       2.434  -2.104   1.063                   C
HETATM   34  C          0       2.904  -0.657   1.074                   C
HETATM   35  H          0       1.992  -2.368   2.031                   H
HETATM   36  H          0       3.274  -2.776   0.869                   H
HETATM   37  H          0       3.731  -0.528   1.777                   H
HETATM   38  H          0       2.089   0.016   1.367                   H
HETATM   39  H          0       0.643  -1.900   0.198                   H
HETATM   40  H          0       2.827  -0.748  -0.860                   H
END
```

S2 (d) EG - Tetramer hybrid model

```
HETATM    1  O          0      -1.793  -0.520   0.481                   O
HETATM    2  O          0      -3.227  -2.779  -0.912                   O
HETATM    3  C          0      -3.178  -0.522   0.096                   C
HETATM    4  C          0      -3.719  -1.940   0.133                   C
HETATM    5  H          0      -3.756   0.095   0.795                   H
HETATM    6  H          0      -3.291  -0.109  -0.912                   H
HETATM    7  H          0      -4.804  -1.896   0.008                   H
HETATM    8  H          0      -3.513  -2.383   1.116                   H
HETATM    9  H          0      -1.405   0.363   0.329                   H
HETATM   10  H          0      -2.280  -2.945  -0.763                   H
HETATM   11  O          0      -1.763   4.095  -1.193                   O
HETATM   12  O          0      -0.469   1.933   0.082                   O
HETATM   13  C          0      -2.054   3.779   0.175                   C
HETATM   14  C          0      -0.868   3.098   0.835                   C
HETATM   15  H          0      -2.333   4.674   0.741                   H
HETATM   16  H          0      -2.910   3.103   0.149                   H
HETATM   17  H          0      -1.128   2.753   1.837                   H
HETATM   18  H          0      -0.018   3.785   0.910                   H
HETATM   19  H          0      -1.201   4.878  -1.221                   H
HETATM   20  H          0      -0.455   2.193  -0.851                   H
HETATM   21  O          0       2.071   0.919   1.078                   O
HETATM   22  O          0       4.214  -0.207  -0.736                   O
HETATM   23  C          0       3.177   1.639   0.528                   C
HETATM   24  C          0       3.642   1.095  -0.817                   C
HETATM   25  H          0       2.932   2.703   0.418                   H
HETATM   26  H          0       3.994   1.554   1.251                   H
HETATM   27  H          0       4.422   1.757  -1.207                   H
HETATM   28  H          0       2.807   1.109  -1.531                   H
HETATM   29  H          0       1.253   1.230   0.656                   H
HETATM   30  H          0       3.509  -0.869  -0.611                   H
HETATM   31  O          0      -0.420  -2.689  -0.396                   O
HETATM   32  O          0       2.350  -2.335  -0.564                   O
HETATM   33  C          0       0.437  -3.357   0.539                   C
HETATM   34  C          0       1.733  -2.581   0.712                   C
```

HETATM	35	H	0	-0.054	-3.482	1.510	H
HETATM	36	H	0	0.635	-4.345	0.118	H
HETATM	37	H	0	2.444	-3.155	1.310	H
HETATM	38	H	0	1.546	-1.623	1.210	H
HETATM	39	H	0	-0.756	-1.851	-0.004	H
HETATM	40	H	0	1.627	-2.147	-1.181	H

Table S3 PDB files of KOH-EG complexes in 1:1, 1:2 and 1:4 ratios for both explicit and hybrid models.

S3 (a) KOH+EG 1:1 ratio explicit model

HETATM	1	O	0	0.450	-1.171	-0.401	O
HETATM	2	O	0	2.422	0.281	1.172	O
HETATM	3	C	0	1.066	0.071	-0.860	C
HETATM	4	C	0	2.444	0.238	-0.249	C
HETATM	5	H	0	1.140	0.060	-1.954	H
HETATM	6	H	0	0.360	0.859	-0.561	H
HETATM	7	H	0	2.874	1.184	-0.589	H
HETATM	8	H	0	3.112	-0.571	-0.593	H
HETATM	9	H	0	0.966	-1.914	-0.734	H
HETATM	10	H	0	1.794	-0.386	1.470	H
HETATM	11	K	0	-2.102	-0.516	0.267	K
HETATM	12	O	0	-1.594	1.654	-0.170	O
HETATM	13	H	0	-1.594	2.608	-0.262	H

S3 (b) KOH+EG 1:1 ratio hybrid model

HETATM	1	O	0	-2.733	-0.056	-0.286	O
HETATM	2	O	0	0.247	-0.026	0.399	O
HETATM	3	C	0	-1.806	-1.127	-0.412	C
HETATM	4	C	0	-0.679	-1.086	0.617	C
HETATM	5	H	0	-2.365	-2.060	-0.284	H
HETATM	6	H	0	-1.360	-1.143	-1.420	H
HETATM	7	H	0	-0.123	-2.029	0.567	H
HETATM	8	H	0	-1.120	-1.014	1.623	H
HETATM	9	H	0	-2.214	0.806	-0.208	H
HETATM	10	H	0	-0.280	0.860	0.299	H
HETATM	11	K	0	2.790	-0.018	-0.174	K
HETATM	12	O	0	-1.172	2.035	-0.019	O
HETATM	13	H	0	-1.377	2.581	0.748	H

S3 (c) KOH+EG 1:2 ratio explicit model

HETATM	1	O	0	-2.293	-1.113	0.110	O
HETATM	2	O	0	-1.126	1.583	0.260	O
HETATM	3	C	0	-3.081	0.070	0.199	C
HETATM	4	C	0	-2.371	1.292	-0.373	C
HETATM	5	H	0	-4.005	-0.096	-0.363	H

HETATM	6	H	0	-3.358	0.258	1.245	H
HETATM	7	H	0	-3.026	2.166	-0.273	H
HETATM	8	H	0	-2.199	1.135	-1.448	H
HETATM	9	H	0	-1.594	-1.032	0.825	H
HETATM	10	H	0	-0.844	0.852	0.891	H
HETATM	11	O	0	2.265	-0.936	0.666	O
HETATM	12	O	0	1.223	1.143	-1.048	O
HETATM	13	C	0	3.028	0.206	0.304	C
HETATM	14	C	0	2.183	1.431	-0.024	C
HETATM	15	H	0	3.730	0.482	1.104	H
HETATM	16	H	0	3.626	-0.078	-0.569	H
HETATM	17	H	0	2.849	2.239	-0.353	H
HETATM	18	H	0	1.661	1.770	0.876	H
HETATM	19	H	0	1.466	-0.684	1.208	H
HETATM	20	H	0	0.365	1.500	-0.731	H
HETATM	21	K	0	0.224	-1.425	-0.946	K
HETATM	22	O	0	-0.217	-0.528	1.563	O
HETATM	23	H	0	-0.301	-0.618	2.516	H

END

S3 (d) KOH+EG 1:2 ratio hybrid model

HETATM	1	O	0	-1.836	-0.969	0.747	O
HETATM	2	O	0	-1.269	1.610	-0.545	O
HETATM	3	C	0	-2.857	-0.143	0.192	C
HETATM	4	C	0	-2.350	0.753	-0.933	C
HETATM	5	H	0	-3.641	-0.792	-0.209	H
HETATM	6	H	0	-3.308	0.474	0.981	H
HETATM	7	H	0	-3.183	1.369	-1.293	H
HETATM	8	H	0	-2.018	0.129	-1.773	H
HETATM	9	H	0	-1.270	-0.350	1.305	H
HETATM	10	H	0	-0.985	1.436	0.411	H
HETATM	11	O	0	1.836	-0.364	1.175	O
HETATM	12	O	0	1.147	0.660	-1.451	O
HETATM	13	C	0	2.753	0.375	0.380	C
HETATM	14	C	0	2.085	1.332	-0.602	C
HETATM	15	H	0	3.444	0.960	1.005	H
HETATM	16	H	0	3.356	-0.355	-0.172	H
HETATM	17	H	0	2.864	1.797	-1.218	H
HETATM	18	H	0	1.574	2.128	-0.052	H
HETATM	19	H	0	1.076	0.219	1.496	H
HETATM	20	H	0	0.261	1.034	-1.249	H
HETATM	21	K	0	0.443	-1.997	-0.513	K
HETATM	22	O	0	-0.357	0.895	1.783	O
HETATM	23	H	0	-0.550	1.328	2.619	H

END

S3 (e) KOH+EG 1:4 ratio explicit model

HETATM	1	O	0	-0.601	-3.271	-0.879	O
HETATM	2	O	0	1.880	-1.947	-0.995	O
HETATM	3	C	0	0.169	-3.068	0.307	C

HETATM	4	C	0	1.037	-1.832	0.176	C
HETATM	5	H	0	-0.475	-2.977	1.192	H
HETATM	6	H	0	0.792	-3.959	0.429	H
HETATM	7	H	0	1.702	-1.676	1.034	H
HETATM	8	H	0	0.405	-0.943	0.069	H
HETATM	9	H	0	-1.329	-2.621	-0.869	H
HETATM	10	H	0	1.319	-2.328	-1.685	H
HETATM	11	O	0	-1.053	3.814	-0.605	O
HETATM	12	O	0	-1.864	1.012	0.476	O
HETATM	13	C	0	-2.313	3.122	-0.736	C
HETATM	14	C	0	-2.634	2.208	0.439	C
HETATM	15	H	0	-3.120	3.852	-0.864	H
HETATM	16	H	0	-2.234	2.535	-1.653	H
HETATM	17	H	0	-3.675	1.887	0.359	H
HETATM	18	H	0	-2.522	2.765	1.381	H
HETATM	19	H	0	-1.161	4.533	0.025	H
HETATM	20	H	0	-0.905	1.210	0.496	H
HETATM	21	O	0	-5.176	-0.000	0.568	O
HETATM	22	O	0	-2.727	-1.444	-0.506	O
HETATM	23	C	0	-5.139	-1.298	-0.034	C
HETATM	24	C	0	-3.821	-2.026	0.188	C
HETATM	25	H	0	-5.963	-1.921	0.340	H
HETATM	26	H	0	-5.292	-1.133	-1.102	H
HETATM	27	H	0	-3.917	-3.050	-0.187	H
HETATM	28	H	0	-3.611	-2.090	1.267	H
HETATM	29	H	0	-5.152	-0.109	1.525	H
HETATM	30	H	0	-2.499	-0.568	-0.131	H
HETATM	31	O	0	0.775	1.810	0.105	O
HETATM	32	O	0	3.286	1.325	-1.133	O
HETATM	33	C	0	2.010	1.996	0.831	C
HETATM	34	C	0	3.127	2.345	-0.137	C
HETATM	35	H	0	1.900	2.791	1.581	H
HETATM	36	H	0	2.251	1.052	1.338	H
HETATM	37	H	0	4.073	2.411	0.406	H
HETATM	38	H	0	2.941	3.311	-0.629	H
HETATM	39	H	0	0.432	2.663	-0.215	H
HETATM	40	H	0	2.395	1.088	-1.425	H
HETATM	41	K	0	4.281	-1.064	-0.200	K
HETATM	42	O	0	3.499	-0.617	1.969	O
HETATM	43	H	0	3.526	-0.643	2.928	H

END

S3 (f) KOH+EG 1:4 ratio hybrid model

HETATM	1	O	0	-1.102	1.822	0.015	O
HETATM	2	O	0	-2.376	3.911	-1.430	O
HETATM	3	C	0	-2.030	2.700	0.657	C
HETATM	4	C	0	-3.034	3.257	-0.341	C
HETATM	5	H	0	-2.557	2.195	1.474	H
HETATM	6	H	0	-1.440	3.517	1.082	H
HETATM	7	H	0	-3.672	4.002	0.141	H
HETATM	8	H	0	-3.676	2.450	-0.717	H

HETATM	9	H	0	-1.506	0.940	-0.084	H
HETATM	10	H	0	-1.675	3.313	-1.720	H
HETATM	11	O	0	3.424	1.349	-0.495	O
HETATM	12	O	0	2.838	1.906	2.288	O
HETATM	13	C	0	2.542	2.412	-0.086	C
HETATM	14	C	0	2.909	2.931	1.294	C
HETATM	15	H	0	2.564	3.231	-0.812	H
HETATM	16	H	0	1.539	1.982	-0.073	H
HETATM	17	H	0	2.203	3.709	1.593	H
HETATM	18	H	0	3.914	3.374	1.274	H
HETATM	19	H	0	4.290	1.723	-0.698	H
HETATM	20	H	0	3.369	1.165	1.972	H
HETATM	21	O	0	-1.784	-2.302	2.067	O
HETATM	22	O	0	0.645	-1.833	0.281	O
HETATM	23	C	0	-0.512	-2.980	2.135	C
HETATM	24	C	0	0.650	-2.109	1.684	C
HETATM	25	H	0	-0.334	-3.328	3.157	H
HETATM	26	H	0	-0.602	-3.850	1.483	H
HETATM	27	H	0	1.583	-2.639	1.890	H
HETATM	28	H	0	0.661	-1.174	2.260	H
HETATM	29	H	0	-1.871	-1.725	2.835	H
HETATM	30	H	0	-0.199	-1.412	0.037	H
HETATM	31	O	0	-4.728	-1.909	-0.160	O
HETATM	32	O	0	-1.987	-0.839	-0.277	O
HETATM	33	C	0	-4.205	-1.179	-1.275	C
HETATM	34	C	0	-2.701	-1.339	-1.419	C
HETATM	35	H	0	-4.690	-1.486	-2.209	H
HETATM	36	H	0	-4.452	-0.132	-1.088	H
HETATM	37	H	0	-2.353	-0.766	-2.281	H
HETATM	38	H	0	-2.442	-2.391	-1.582	H
HETATM	39	H	0	-4.689	-2.851	-0.361	H
HETATM	40	H	0	-2.217	-1.362	0.517	H
HETATM	41	K	0	2.816	-1.229	-1.313	K
HETATM	42	O	0	4.759	-2.623	-1.902	O
HETATM	43	H	0	5.517	-3.170	-2.130	H

Table S4 Comparison of IR vibrational modes of EG monomer, dimer, and tetramer for both explicit (EXC) and hybrid (HYB) model.

Experimental IR frequency (cm^{-1})	Calculated IR scaled frequency (cm^{-1})						Vibrational Modes
	Monomer		Dimer		Tetramer		
	EXC	HYB	EXC	HYB	EXC	HYB	
3564.25, 3446.09,	3615.36, 3587.69	3602.98, 3582.17	3624.05	3608.49	3626.38, 3558.16	3609.22, 3550.60	υ(OH)
3417.96					3546.39		υ(OH) + υ(OH) [H-bonding]
3395.85, 3354.19, 3311.24, 3300.52, 3296.23			3499.68, 3490.68, 3379.52	3452.44, 3440.67, 3392.26	3559.13, 3488.71, 3461.84, 3432.65, 3340.48	3550.45, 3467.94 3441.47, 3398.40 3382.50, 3260.35	υ(OH) [H-bonding]
2931.66, 2923.48, 2924.35, 2880.50, 2876.49, 2875.27, 2873.34	2963.26, 2939.95	2963.07, 2951.54	2970.06, 2967.70 2940.65, 2932.48	2971.44, 2962.48, 29,5.52, 2943.29	2975.70, 2965.03, 2963.15, 2953.91, 2949.03, 2939.37, 2919.40, 2913.96	2981.36, 2970.01, 2968.71, 2957.28, 2954.81, 2944.65, 2930.28, 2925.95	υ_a(CH)
2856.76, 2841.65 2836.08, 2827.60, 2738.76, 2728.04, 2721.61	2884.39, 2836.13	2900.72, 2867.68	2894.95, 2878.35, 2856.52, 2839.25	2911.96, 2891.88, 2885.74, 2871.13	2909.47, 2902.38, 2898.16, 2891.44, 2870.41, 2865.40, 2849.42, 2832.41	2916.96, 2909.15, 2907.66, 2901.45, 2884.96, 2881.96, 2879.44, 2868.34	υ_S(CH)
1455.87, 1455.15, 1440.14, 1434.42, 1427.27, 1417.26,	1475.02, 1466.91	1469.44, 1461.15	1471.56, 1467.14, 1466.93, 1465.13	1469.24, 1464.24, 1463.13, 1459.99	1492.88, 1481.96, 1475.98, 1474.26, 1471.83, 1463.06, 1461.92, 1458.68	1483.41, 1475.76, 1471.53, 1467.11, 1466.12, 1461.69, 1458.36, 1456.75	χ(CH_2)
1372.24, 1367.24			1448.35	1429.62	1441.22, 1433.95	1440.01, 1431.06	β(COH)
1410.83, 1386.53	1405.58, 1378.17, 1350.55	1400.67, 1377.01, 1346.99	1395.96, 1385.96 1378.23, 1373.14	1386.05, 1375.81, 1372.94	1385.20, 1381.45, 1379.18, 1372.44, 1371.48	1386.48, 1380.96, 1375.65, 1375.12	ω(CH_2)
1403.69, 1372.87,	1346.42	1353.27	1407.81, 1360.83	1406.11, 1394.52	1421.19, 1411.39, 1409.83	1409.43, 1408.19, 1404.62, 1371.63	β(COH) +ω(CH_2) [Association bands]
1368.85, 1361.88, 1342.98, 1342.22, 1338.67, 1337.98, 1335.79, 1330.51, 1268.89				1360.12	1393.86, 1392.04, 1343.02	1398.04, 1392.87, 1346.29, 1334.82, 1317.96, 1223.00, 1211.74, 1200.95, 1193.53, 1181.85, 1043.79	γ(CH2) + β (COH) [Association bands]
1258.60, 1254.32, 1208.63, 1205.77, 1204.99	1217.00, 1181.34	1222.40, 1175.77	1354.71, 1261.66, 1256.68, 1193.97, 1187.59, 1066.27, 1046.88	1356.81, 1260.36, 1255.57, 1195.88, 1189.09, 1062.83	1352.52, 1338.29, 1323.00, 1266.22, 1260.63, 1234.63, 1215.36, 1209.57, 1204.12, 1195.49, 1185.51	1356.73, 1262.23, 1260.18, 1236.69, 1093.21, 1084.78	γ(CH2)

1086.33, 1084.16, 1082.80, 883.40, 881.95, 862.66,	1083.30, 1039.45, 857.49	1066.19, 1023.51, 855.43	1095.80, 1091.94, 1053.75, 846.29, 842.20	1076.58, 1071.12, 1044.55, 1030.20, 874.82, 849.24, 845.01	1100.90, 1079.82, 1071.36, 891.78, 878.80, 873.41, 1033.49, 852.94, 842.26	1079.38, 1073.19, 1062.28, 1060.30, 1057.76, 1045.27, 1039.10, 1027.98, 1022.98, 873.57, 870.03, 852.81, 847.63	ν(CO)+ν(CC)
				1041.57	1102.45	884.13	γ(CH_2) +ν(CO) [Association bands]
859.81, 746.15, 749.01					1064.59, 1063.37, 1042.63		ν(CO)+ν(CC)+ γ(CH_2) [Association bands]
861.22	1045.97, 876.00	1043.25, 873.27,	1036.25, 887.79, 882.60	879.22	1052.70, 1048.08, 903.05		ρ(CH_2)
723.28, 716.85						901.04	ρ(CH_2) + β (COH) [Association bands]
						866.13, 863.12	ρ(CH_2) +υ(CO) [Association bands]
1034.18, 1032.78					1050.38, 870.06, 859.08		υ(CO)
698.98, 646.10, 636.09, 623.94, 617.51	451.72	399.52	792.17, 643.65, 581.83	786.65, 645.68, 546.84	820.54, 716.03, 683.44, 650.21, 583.42, 494.18, 332.49	322.41, 838.00, 706.61, 687.77, 622.38, 620.03, 469.51, 357.45, 322.41	δ(OH) deformation [H-bonding]
614.65 610.36	535.50	533.59	520.58, 508.00, 344.36, 335.79	522.21, 511.50, 340.04	557.14, 546.76, 528.27, 500.49	552.05, 542.68, 520.71	β(CCO)
602.50	325.04	318.73	332.64, 317.27	313.36	333.58, 509.50, 355.69, 346.79, 332.73	511.02, 503.30, 345.74, 343.91	δ (CCO)
	302.39	309.21	313.36	317.27			τ(OH)
			262.09, 213.67	332.64, 317.27	304.134, 257.80, 222.25, 210.82	333.58, 262.71, 217.02, 212.95	τ(CC)
	160.11	149.11	179.32, 143.14, 121.26, 93.92, 64.28, 46.62	182.65, 138.89, 123.25, 92.98, 80.50, 49.30	195.20, 170.49, 155.18, 129.24, 116.82, 109.39, 103.65, 94.01, 79.04, 69.87, 64.41, 60.22, 51.77, 45.05, 38.20, 32.15, 25.10, 13.26, 9.42	195.33, 167.57, 160.43, 133.86, 112.74, 112.55, 102.33, 88.30, 82.33, 76.53, 67.87, 62.19, 59.01, 49.24, 38.26, 35.55, 28.89, 23.97, 13.11	Lattice modes

Types of vibration: υ, stretching; as, asymmetric; s, symmetric; β, bending; χ, scissoring: δ, deformation; oop, out-of-plane bending; ω, wagging; γ, twisting; ρ, rocking; τ, torsion, puck, puckering.

Table S5 Comparison of IR modes of ~~KOH-EG complex for~~ KOH-EG complexes 1:1, 1:2 and 1:4 ratio for both explicit (EXC) and hybrid (HYB) model.

Experimental IR frequency (cm^{-1})	Calculated IR scaled frequency (cm^{-1})						Vibrational Modes
	KOH+EG (1:1)		KOH+EG (1:2)		KOH+EG (1:4)		
	EXC	HYB	EXC	HYB	EXC	HYB	
3396.50	3690.999	3632.149	3661.85	3652.36	3673.21	3640.10	ν(OH) [OH^- ion]
3364.43, 3355.71, 3347.21, 3340.32	3614.60, 3611.44	2840.35	3298.00	3261.86	3627.00, 3616.86, 3570.79, 3564.73	3611.60, 3609.54 3607.22, 3593.32 3575.81	ν(OH)
3306.24					3462.11	3432.56, 3355.08	ν(OH)+ ν(OH)[H-bonding]
3327.68, 3313.39, 3300.52			3065.20, 2811.21	2910.85, 2759.92 2685.54	3410.54, 3361.29, 3322.75	3395.23	ν(OH)[H-bonding]
2940.19		2940.28, 2923.06	2898.27, 2913.88,	2940.28, 2923.06			ν(OH)[H-bonding] + ν_{as}(CH)
2937.45, 2936.72, 2912.34, 2876.63, 2765.54, 2758.57	2946.77, 2917.49	2905.05, 2775.91, 2316.52	2933.65, 2907.67	2938.20, 2937.80	2975.66, 2968.46, 2961.62, 2960.01, 2947.46, 2936.98, 2922.70,2913.95	2976.34, 2973.89, 2969.47, 2960.69, 2957.56, 2955.99, 2952.51, 2946.17	ν_{as}(CH)
2738.81, 2735.20, 2727.32, 2723.04, 2719.51, 2714.46, 2698.12	2842.27, 2816.04		2880.26, 2877.19, 2857.52		2900.22, 2884.48, 2877.15, 2873.89, 2861.48, 2854.48, 2851.57, 2849.96	2916.53, 2907.47, 2899.96, 2894.89, 2893.47, 2882.14, 2875.88, 2873.28	ν_{s}(CH)
2873.13, 2709.41			2850.51	2886.16, 2873.72, 2864.37			ν_{s}(CH)+ ν(OH)[H-bonding]
1422.76		1587.68, 1499.58	1568.61, 983.69, 956.80, 732.87	1548.90, 1011.34, 973.81, 711.97,	1434.62, 1413.92, 1355.18		β(COH)
1456.56, 1455.15, 1435.24, 1428.37,	1507.40, 1475.82, 1447.97	1461.64, 1457.98	1482.88, 1475.08, 1468.49	1475.332	1512.38, 1506.36, 1478.31, 1476.57, 1467.93, 1465.51, 1461.81	1473.54, 1473.39, 1470.30, 1467.25, 1464.43, 1461.75, 1461.06, 1458.04	χ(CH2)
1417.27, 1415.83, 1339.39, 1338.69, 1337.93, 1336.48	1399.67	1373.63, 1355.20	1380.23, 1378.17, 1369.40, 1360.47	1380.33, 1378.30 1373.56, 1359.56	1447.78, 1426.52, 1396.28, 1391.48, 1388.02, 1382.48, 1373.43, 1370.48	1389.52, 1383.25	ω(CH2)
1416.63, 1410.71, 1335.82			1502.17, 1485.47, 1465.92, 1438.11	1498.72, 1487.95, 1465.73, 1462.34, 1458.73, 1449.25	1470.31, 1468.52	1433.61	χ(CH2)+ β(COH) [Association bands]
1404.55, 1399.12, 1372.43, 1367.20, 1259.32, 1034.88					1381.03	1412.60, 1407.02, 1402.07, 1396.06, 1379.96, 1374.31, 1372.10, 1369.85,	ω(CH2)+ β(COH) [Association bands]

						1360.17	
1344.36, 1259.32 1343.65, 1254.76, 1213.97, 1207.86	1340.72, 1337.45, 1214.52, 1187.39, 1051.91	1269.80, 1232.47, 1093.14	1275.70, 1268.33, 1249.79, 1236.72, 1115.15	1278.26, 1271.16 1248.80, 1236.53, 1100.022	1233.30, 1211.06, 1198.60, 1187.24, 1183.45, 1086.23, 1082.50, 1036.07	1075.58, 1049.43	γ(CH2)
1365.08, 1364.38, 1363.68, 1362.22, 1085.60, 1039.17			1071.49	1117.60, 1066.75	1352.39, 1344.60, 1343.45, 1273.56, 1263.97, 1243.77		γ(CH2) + β(COH) [Association bands]
			1107.55			1045.09	γ(CH2) +ν(CO) [Association bands]
1168.37, 1161.97, 1154.25, 1149.97			1062.49, 1061.78	1049.91, 1046.70	1061.48, 1059.71, 1046.38, 1043.47 1020.83	1018.36	ν(CO)
1084.21, 1082.76	1084.992	1138.13, 950.88					β(COH)
1038.47, 1037.77, 1036.33, 1035.63, 1033.48, 1023.00, 861.22, 821.75	1000.66, 835.043	1080.63, 1063.49, 846.28	1095.10, 1084.31 868.35, 857.24	1081.43, 1080.15 863.46, 854.76	1097.09, 1090.87, 1065.04, 1029.48, 873.25, 867.26, 864.34, 841.60	1073.77, 1072.02, 1066.80, 1064.76, 873.05, 861.25, 859.58, 853.67, 842.75	ν(CC)+ν(CO)
881.94, 803.18, 799.74, 796.90	884.473	884.930	873.56, 871.80	871.13, 869.99	890.61, 887.88, 883.41, 855.90	882.95, 876.99, 873.69	ρ(CH2)
638.95, 628.22, 622.51	549.684	520.820, 407.446	544.81, 528.65, 372.95	543.08	553.73, 547.43, 523.47, 507.57	536.28, 533.96, 530.72, 513.60	β(CCO)
778.32, 774.03, 748.30, 736.86	429.144, 423.664, 379.928, 363.578	391.97, 366.64, 249.91	443.64, 265.16, 242.69	708.64, 368.79, 265.17	793.05, 748.24 734.88	774.059, 688.60, 336.59, 335.84, 302.37, 297.80, 247.94	τ(OH)
723.67, 667.53 644.67			551.77, 528.65	461.37, 377.24	519.23, 493.00, 395.78, 358.24, 329.04, 286.51	588.81, 416.09	δ(OH)
	343.27, 303.73	317.08	334.43, 323.27 223.80	537.07, 329.42 316.13, 238.11	364.18, 351.97, 348.45, 344.93, 322.02, 269.92	391.34, 351.07, 343.55, 321.42, 309.93	δ(CCO)
	173.639	219.33	217.38, 208.38		245.90, 235.68, 223.73	230.94	τ(CC)

	151.718, 124.570, 106.297, 58.832, 36.405	131.798, 83.848, 26.864, 18.841	173.96, 155.03, 144.09, 117.50, 101.77, 78.31, 71.88, 46.31	197.26, 155.68, 138.92, 121.99, 113.33, 95.04 87.00, 64.55, 51.80	189.58, 162.21, 149.82, 137.82, 129.41, 128.43, 118.34, 111.51, 107.13, 95.41, 91.65, 85.91, 80.05, 69.63, 61.76, 60.24, 49.73, 46.65, 41.65, 34.29, 22.10, 21.04, 14.11	183.59, 159.99, 143.89, 136.95, 127.80, 113.81, 93.49, 89.11, 76.50, 70.16, 68.69, 56.64, 51.71, 43.40, 41.10, 37.93, 35.72, 31.65, 27.28, 24.39, 22.26, 18.95, 16.27, 15.73, 11.09	Lattice modes

Types of vibration: υ, stretching; as, asymmetric; s, symmetric; β, bending; χ, scissoring: δ, deformation; oop, out-of-plane bending; ω, wagging; γ, twisting; ρ, rocking; τ, torsion, puck, puckering

Table S6 (a) A comparison of second order perturbation theory analysis of Fock matrix in NBO Basis ~~(hydrogen bonds shaded yellow)~~ of explicit and hybrid models for EG monomer and dimer.

Explicit model					Hybrid model				
Donor NBO (i)	Acceptor NBO(j)	$E^{(2)a}$ kJ/mol	E(j)-E(i)[b]	F(i,j)[c]	Donor NBO (i)	Acceptor NBO(j)	$E^{(2)a}$ kJ/mol	E(j)-E(i)[b]	F(i,j)[c]
**Monomer**									
σ C 3 - H 5	σ*O 2 - C 4	16.57	0.82	0.051	σ C 3 - H 5	σ*O 2 - C4	17.62	0.80	0.052
σ C 4 - H 7	σ*O1 - C 3	18.24	0.78	0.052	σ C 4 - H 7	σ*O 1 - C3	18.54	0.78	0.053
LP (2) O1	σ*C3 - C 4	25.36	0.69	0.058	LP (2) O 1	σ*C 3 - C4	25.19	0.70	0.058
LP (π2) O 1	σ*C3 - H 5	17.53	0.69	0.048	LP (2) O 1	σ*C 3 - H5	16.99	0.70	0.048
LP (2) O 2	σ*C 3 - C 4	15.77	0.67	0.045	LP (2) O 2	σ*C 3 - C4	17.32	0.69	0.048
LP (2) O 2	σ*C4 - H 8	34.90	0.66	0.067	LP (2) O 2	σ*C 4 - H8	29.59	0.69	0.062
**Dimer**									
σ (1) C 3 - H 5	σ*O2 - C 4	17.70	0.83	0.053	σ C 3 - H 5	σ*(1) O 2 - C 4	18.95	0.8	0.054
σ (1) C 4 - H 7	σ*O1 - C 3	20.88	0.77	0.055	σ (1) C 4 - H 7	σ*(1) O 1 - C 3	20.59	0.78	0.055
LP (π) O 1	σ*C3 - C 4	18.37	0.73	0.05	LP (2) O 1	σ*(1) C 3 - C 4	16.07	0.74	0.048
LP (π) O 1	σ*C3 - H 5	20.21	0.72	0.053	LP (2) O 1	σ*(1) C 3 - H 5	19.46	0.75	0.053
LP (2) O 2	σ*C 3 - C 4	18.87	0.69	0.05	LP (2) O 2	σ*(1) C 4 - H 8	27.15	0.72	0.062
LP (2) O 2	σ*C4 - H 8	30.29	0.68	0.063	LP (2) O 1	σ*(1) O 11 - H 19	24.02	0.8	0.061
LP (2) O 1	σ*O11 - H 19	17.57	0.79	0.052	LP (2) O 2	σ*(1) O 12 - H 20	29.08	0.79	0.067
LP (2) O 2	σ*O12 - H 20	21.26	0.79	0.057	LP (2) O 11	σ*(1) O 2 - H 10	37.53	0.78	0.075
LP (2) O 11	σ*O2 - H 10	43.30	0.79	0.081	σ (1) C 13 - H 15	σ*(1) O 12 - C 14	19.12	0.8	0.054
σ (1) C 13 - H 15	σ*O12 - C14	18.20	0.82	0.053	σ (1) C 14 - H 17	σ*(1) O 11 - C 13	20.59	0.79	0.056
σ (1) C 14 - H 17	σ*O11 - C13	20.46	0.78	0.055	LP (2) O 11	σ*(1) C 13 - C 14	13.22	0.73	0.043
LP (2) O 11	σ*C13 - H15	26.03	0.74	0.061	LP (2) O 11	σ*(1) C 13 - H 15	25.52	0.74	0.06
LP (2) O 12	σ*C13 - C14	28.83	0.67	0.061	LP (2) O 12	σ*(1) C 13 - C 14	27.70	0.68	0.06
LP (2) O 12	σ*C14 - H18	26.07	0.66	0.057	LP (2) O 12	σ*(1) C 14 - H 18	21.17	0.68	0.053

[a]E(2) means energy of hyper conjugative interaction (stabilization energy).

[b]Energy difference between donor and acceptor i and j NBO orbitals.

[c]F(i, j) is the Fock matrix element between i and j NBO orbit

Table S6 (b) A comparison of second order perturbation theory analysis of Fock matrix in NBO Basis ~~(hydrogen bonds shaded yellow)~~ of explicit and hybrid model for KOH+EG in the ratio 1:1, 1:2.

Explicit Model					Hybrid model				
**Donor NBO (i)**	**Acceptor NBO(j)**	**$E^{(2)}$[a] kJ/mol**	**E(j)-E(i)[b]**	**F(i,j)[c]**	**Donor NBO (i)**	**Acceptor NBO(j)**	**E(2)[a] kJ/mol**	**E(j)-E(i)[b]**	**F(i,j)[c]**
**KOH+EG (1:1)**									
σC3 - H5	σ*O2 - C4	18.79	0.8	0.054	σ C3 - H5	σ*(σ)O2 - C4	19.29	0.81	0.054
σC4 - H7	σ*O1 - C3	20.71	0.75	0.054	σ C4 - H7	σ*(σ)O1 - C3	18.79	0.82	0.054
LP (2) O1	σ*C3 - C4	18.74	0.73	0.051	LP (2) O1	σ*C3 - C4	17.91	0.67	0.048
LP (2) O1	σ*C3 - H5	17.66	0.71	0.049	LP (2) O1	σ*C3 – H6	35.02	0.66	0.067
LP (2) O2	σ*C3 – C4	13.01	0.68	0.041	LP (2) O2	σ*C3 – C4	19.50	0.67	0.050
LP (2) O2	σ*C4 - H8	35.86	0.66	0.07	LP (2) O2	σ*C3 – H8	31.67	0.67	0.064
LP (3) O12	σ*C3 - H6	31.21	0.66	0.063	σ*O2 – H10	σ*O1 - H9	13.98	0.04	0.031
					LP (2) O12	σ*O1 - H9	103.68	0.69	0.117
					LP (2) O12	σ*O2 – H10	14.02	0.65	0.042
					LP (3) O12	σ*O1 - H9	56.44	0.91	0.100
					LP (2) O12	σ*O2 – H10	201.63	0.87	0.184
**KOH+EG (1:2)**									
σC3 – H5	σ *O2 – C4	19.54	0.80	0.055	σC3 – H5	σ *O2 – C4	19.58	0.80	0.054
σC4 - H7	σ *O1 - C3	18.83	0.80	0.054	σC4 – H7	σ *O1 – C3	19.12	0.80	0.054
LP (2) O1	σ *C3 – C4	25.31	0.69	0.058	LP (2) O 1	σ *C3 - C4	28.54	0.68	0.061
LP (2) O2	σ *C3 – H6	19.00	0.70	0.051	LP (2) O 1	σ *C3 – H6	17.28	0.68	0.048
LP (1) O2	σ *C4 – H7	14.39	0.91	0.050	LP (2) O 2	σ *C4 - H8	27.82	0.74	0.063
LP (2) O2	σ *C4 – H8	28.83	0.74	0.064	LP (2) O 2	σ *O12 – H20	68.62	0.79	0.103
LP (2) O2	σ *O12 – H20	58.53	0.79	0.095	σ C13 - H15	σ *O15 - C14	19.16	0.79	0.054
σC14 – H17	σ *O11 – C13	19.12	0.79	0.054	σ C14 - H17	σ *O11 - C13	19.29	0.81	0.055
σC14 – H17	σ *O11 – C13	19.67	0.80	0.055	LP (2) O 11	σ *C13– C14	15.61	0.68	0.045
LP (2) O11	σ*C13 – H15	35.19	0.70	0.069	LP (2) O 11	σ *C13– H15	35.10	0.68	0.068
LP (2) O12	σ*C13 - C14	15.52	0.70	0.046	LP (2) O 12	σ *C13– C14	23.31	0.69	0.055
LP (2) O12	σ*C14 - H 17	28.33	0.71	0.062	LP (2) O 12	σ *C13– H17	21.97	0.69	0.054
LP (1) O22	σ*O2 – H10	18.45	0.74	0.051	LP (1) O 22	σ *O1– H9	21.30	0.73	0.055
LP (2) O22	σ*O1 - H9	16.32	0.84	0.051	LP (1) O 22	σ *O2– H10	53.30	0.72	0.086
LP (2) O22	σ*O2 – H10	31.92	0.86	0.072	LP (2) O 22	σ *O1– H9	46.11	0.94	0.091
LP (2) O22	σ*O11 – H19	8.58	0.84	0.051	LP (3) O 22	σ *O1– H9	79.66	0.85	0.114
LP (2) O22	σ*O11 – H19	11.76	0.86	0.072	LP (2) O 22	σ *O2– H10	117.40	0.84	0.138
					LP (1) O 22	σ *O11– H19	28.12	0.74	0.063
					LP (1) O 22	σ *O11– H19	117.70	0.94	0.145

[a]E(2) means energy of hyper conjugative interaction (stabilization energy).
[b]Energy difference between donor and acceptor i and j NBO orbitals.
[c]F(i, j) is the Fock matrix element between i and j NBO orbit.

Table S6 (c) A comparison of second order perturbation theory analysis of Fock matrix in NBO Basis ~~(hydrogen bonds shaded yellow)~~ of explicit and hybrid model for EG tetramer and EG+KOH in the ratio 1:4.

EG tetramer					KOH+EG (1:4)				
Donor NBO (i)	Acceptor NBO(j)	$E^{(2)a}$ kJ/mol	E(j)-E(i)[b]	F(i,j)[c]	Donor NBO (i)	Acceptor NBO(j)	$E^{(2)a}$ kJ/mol	E(j)-E(i)b	F(i,j)c
**Explicit model**									
σ C 3 - H 5	σ*O 2 - C 4	17.87	0.83	0.053	σ (1) C 3 - H 5	σ*(1) O 2 - C 4	17.78	0.78	0.051
σ C 4 - H 7	σ*O 1 - C 3	18.74	0.77	0.053	LP (2) O 1	σ*(1) C 3 - C 4	25.15	0.69	0.058
LP (1) O 1	σ*C 3 - H 6	17.53	0.88	0.054	LP (2) O 1	σ*(1) C 3 - H 5	18.87	0.68	0.05
LP (2) O 1	σ*C 3 - H 5	21.13	0.81	0.058	LP (2) O 2	σ*(1) C 4 - H 8	27.03	0.7	0.06
LP (2) O 2	σ*C 3 - C 4	32.84	0.67	0.065	σ (1) C 13 - H 15	σ*(1) O 12 - C 14	17.95	0.82	0.053
LP (2) O 2	σ*C 4 - H 8	18.79	0.66	0.049	σ (1) C 14 - H 17	σ*(1) O 11 - C 13	20.67	0.76	0.055
LP (2) O 1	σ*O31 - H 39	54.60	0.87	0.096	LP (2) O 11	σ*(1) C 13 - H 15	18.87	0.75	0.052
LP (1) O 12	σ*O 1 - H 9	16.76	1.09	0.059	LP (1) O 12	σ*(1) C 13 - C 14	23.93	0.81	0.061
LP (2) O 12	σ*O 1 - H 9	26.82	0.81	0.065	LP (2) O 12	σ*(1) C 14 - H 18	21.67	0.82	0.059
σ C 13 - H 15	σ*O 12 - C 14	16.90	0.79	0.05	LP (2) O 12	σ*(1) O 22 - H 30	46.11	0.91	0.09
σ C 14 - H 17	σ*O 11 - C 13	16.74	0.79	0.05	LP (2) O 11	σ*(1) O 31 - H 39	27.57	0.82	0.066
LP (2) O 11	σ*C 13 - C 14	26.23	0.69	0.059	σ (1) C 23 - H 25	σ*(1) O 22 - C 24	18.24	0.82	0.053
LP (2) O 12	σ*C 14 - H 18	28.33	0.73	0.063	σ (1) C 24 - H 27	σ*(1) O 21 - C 23	19.92	0.78	0.055
σ C 23 - H 25	σ*O 22 - C 24	17.11	0.84	0.052	LP (2) O 21	σ*(1) C 23 - C 24	23.31	0.69	0.056
σ C 24 - H 27	σ*O 21 - C 23	20.25	0.79	0.055	LP (2) O 21	σ*(1) C 23 - H 25	23.14	0.68	0.055
LP (2) O 21	σ*C 23 - C 24	27.91	0.69	0.061	LP (1) O 22	σ*(1) C 24 - H 28	17.53	0.88	0.054
LP (2) O 21	σ*C 23 - H 25	17.24	0.67	0.047	LP (2) O 22	σ*(1) C 23 - C 24	28.12	0.75	0.064
LP (2) O 21	σ*C 34 – H38	3.85	1.00	0.027	LP (2) O 31	σ*(1) O 12 - H 20	45.52	0.81	0.085
LP (2) O 22	σ*C 23 - C 24	33.01	0.66	0.065	σ (1) C 33 - H 35	σ*(1) O 32 - C 34	18.12	0.78	0.052
LP (2) O 22	σ*C 24 - H 28	20.59	0.65	0.051	σ (1) C 34 - H 37	σ*(1) O 31 - C 33	17.91	0.79	0.052
LP (2) O 31	σ*O 2 - H 10	21.51	0.79	0.058	LP (2) O 31	σ*(1) C 33 - C 34	22.22	0.77	0.057
LP (2) O 32	σ*O 22 - H 30	41.42	0.84	0.082	LP (2) O 32	σ*(1) C 34 - H 38	30.88	0.69	0.064
σ C 34 - H 37	σ*O 31 - C 33	16.99	0.79	0.051	LP (3) O 42	σ*(1) C 4 - H 7	12.43	0.81	0.044
LP (2) O 31	σ*C 33 - C 34	26.11	0.71	0.059	LP (3) O 42	σ*(1) C 33 - H 36	26.61	0.68	0.059
LP (2) O 32	σ*C 34 - H 38	21.17	0.76	0.056	LP (1) O 42	LP*(1) K 41	27.95	0.72	0.062
					LP (2) O 42	LP*(1) K 41	18.79	0.63	0.047
**Hybrid model**									
σ (1) C 3 - H 5	σ*O 2 - C 4	19.46	0.8	0.054	σ (1) C 3 - H 5	σ*(1) O 2 - C 4	17.15	0.8	0.051
σ (1) C 4 - H 7	σ*O 1 - C 3	18.45	0.78	0.052	σ (1) C 4 - H 7	σ*(1) O 1 - C 3	17.41	0.8	0.052
LP (1) O 1	σ*C 3 - H 6	18.37	0.86	0.055	LP (2) O 1	σ*(1) C 3 - C 4	27.95	0.69	0.061
LP (2) O 1	σ*C 3 - H 5	19.08	0.84	0.056	LP (1) O 1	σ*(1) C 13 – H16	3.52	0.99	0.026
LP (2) O 2	σ*C 3 - C 4	28.08	0.69	0.061	LP (2) O 2	σ*(1) C 4 - H 8	30.59	0.69	0.063
LP (2) O 2	σ*C 4 - H 8	20.08	0.68	0.051	σ (1) C 13 - H 15	σ*(1) O 12 - C 14	17.99	0.8	0.052
LP (2) O 1	σ*O 31 - H 39	68.28	0.88	0.107	σ (1) C 14 - H 17	σ*(1) O 11 - C 13	19.16	0.78	0.053

LP (1) O 12	σ*O 1 - H 9	19.41	1.08	0.063	LP (2) O 11	σ*(1) C 13 - C 14	23.51	0.71	0.056
LP (2) O 12	σ*O 1 - H 9	33.77	0.79	0.072	LP (2) O 11	σ*(1) C 13 - H 15	16.99	0.71	0.048
σ C 13 - H 15	σ*O 12 - C 14	17.99	0.78	0.052	LP (2) O 12	σ*(1) C 13 - C 14	18.28	0.69	0.049
σ C 14 - H 17	σ*O 11 - C 13	17.28	0.79	0.051	LP (2) O 12	σ*(1) C 14 - H 18	28.79	0.69	0.061
LP (2) O 12	σ*C 14 - H 18	26.49	0.73	0.061	σ (1) C 23 - H 25	σ*(1) O 22 - C 24	18.79	0.8	0.054
LP O 12	σ*O 21 - H 29	21.09	1.09	0.066	σ (1) C 24 - H 27	σ*(1) O 21 - C 23	20.63	0.77	0.055
LP O 12	σ*C34-H38	2.93	0.98	0.023	LP (2) O 21	σ*(1) C 23 - H 25	16.95	0.78	0.051
σ C 23 - H 25	σ*O 22 - C 24	18.58	0.81	0.054	LP (2) O 22	σ*(1) C 23 - C 24	22.47	0.69	0.055
σ C 24 - H 27	σ*O 21 - C 23	19.71	0.8	0.055	LP (2) O 22	σ*(1) C 24 - H 28	25.36	0.69	0.058
LP (2) O 21	σ*C23 - C 24	28.62	0.69	0.061	LP (2) O 21	σ*(1) O 32 - H 40	39.75	0.82	0.079
LP (2) O 22	σ*C23 - C 24	29.59	0.68	0.062	LP (1) O 32	σ*(1) O 1 - H 9	41.84	1.05	0.092
LP (2) O 22	σ*C24 - H 28	17.57	0.68	0.048	LP (2) O 32	σ*(1) O 22 - H 30	36.49	0.78	0.074
LP (2) O 31	σ*O 2 - H 10	31.80	0.79	0.07	σ (1) C 33 - H 35	σ*(1) O 32 - C 34	19.25	0.79	0.054
LP (2) O 32	σ*O22 - H 30	45.14	0.84	0.085	σ (1) C 34 - H 37	σ*(1) O 31 - C 33	19.00	0.79	0.054
σ C 33 - H 35	σ*O 32 - C 34	17.20	0.79	0.051	LP (2) O 31	σ*(1) C 33 - C 34	26.23	0.69	0.059
σ C 34 - H 37	σ*O 31 - C 33	17.15	0.79	0.051	LP (2) O 31	σ*(1) C 33 - H 35	18.58	0.69	0.05
LP (2) O 31	σ*C 33 - C 34	25.19	0.72	0.059	LP (2) O 32	σ*(1) C 33 - C 34	23.60	0.72	0.057
LP (2) O 32	σ*C 34 - H 38	19.54	0.77	0.054	LP (3) O 42	LP*(2) K 41	31.80	0.96	0.076

[a]E(2) means energy of hyper conjugative interaction (stabilization energy).
[b]Energy difference between donor and acceptor i and j NBO orbitals.
[c] F(i, j) is the Fock matrix element between i and j NBO orbitals.

Table S7 (a) A comparison of the electron density (ρ_{BCP}), Laplacian of the electron density ($\nabla^2 \rho_{BCP}$), the Lagrangian kinetic energy (G_{BCP}), the potential electron density (V_{BCP}), the energy density (H_{BCP}) and the binding energy (BE_{BCP}) for EG oligomers (dimer and tetramer) for both explicit (EXC) and hybrid (HYB) model for all possible intermolecular interactions.

Bonds	Bond length (Å)		ρ_{BCP} (a.u.)		$\nabla^2 \rho_{BCP}$		G_{BCP}		V_{BCP}		H_{BCP}		BE_{BCP} (kcal/mol)	
	EXC	HYB	EXC	HYB	EXC	HYB	EXC	HYB	EXC	HYB	EXC	HYB	EXC	HYB
**Dimer**														
**H-bonds**														
O1H19	1.9850	1.9420	0.0238	0.0261	0.0854	0.0923	0.0196	0.0215	-0.0178	-0.0200	0.0018	0.0015	-4.5670	-5.0801
O2H20	1.9830	1.9370	0.0227	0.0254	0.0815	0.0900	0.0184	0.0207	-0.0164	-0.0020	0.0017	0.0017	-4.3216	-4.9239
O11H10	1.8640	1.8970	0.0317	0.0294	0.1055	0.0989	0.0260	0.0239	-0.0257	-0.0231	0.0003	0.0008	-6.3293	-5.8163
**Van der Waals interactions**														
H6H18	2.9610	2.9330	0.0018	0.0018	0.0065	0.0067	0.0012	0.0013	-0.0008	-0.0009	0.0004	0.0004	0.3408	0.3408
**Tetramer**														
**H-bonds**														
O1H39	1.7920	1.7560	0.0357	0.0394	0.1230	0.1295	0.0309	0.0338	-0.0310	-0.0353	-0.0014	-0.0014	-7.2217	-8.0471
O12H9	1.8830	1.8440	0.0282	0.0312	0.1028	0.1092	0.0241	0.0265	-0.0224	-0.0256	0.0017	0.0008	-5.5486	-6.2178
O12H29	2.0590	1.9460	0.0198	0.0251	0.0711	0.0894	0.0159	0.0205	-0.0139	-0.0187	0.0019	0.0018	-3.6747	-4.8570
O31H10	1.9340	1.9130	0.0251	0.0270	0.0926	0.0957	0.0213	0.0224	-0.0194	-0.0209	0.0019	0.0015	-4.8570	-5.2809
O32H30	1.8770	1.8690	0.0291	0.0300	0.1023	0.1037	0.0243	0.0249	-0.0230	-0.0238	0.0013	0.0011	-5.7493	-5.9501
O21H38	2.5070	2.6000	0.0093	0.0078	0.0278	0.0231	0.0062	0.0052	-0.0054	-0.0046	0.0008	0.0006	-1.3323	-0.9977
**Van der Waals interactions**														
H5H16		3.1960		0.0012		0.0044		0.0008		-0.0005		0.0003		0.4746

Table S7 (b) A comparison of the electron density (ρ_{BCP}), Laplacian of the electron density ($\nabla^2\rho_{BCP}$), the Lagrangian kinetic energy (G_{BCP}), the potential electron density (V_{BCP}), the energy density (H_{BCP}) and the binding energy (BE_{BCP}) for KOH-EG complexes for all ratios for both explicit (EXC) and hybrid (HYB) model for all possible intermolecular interactions.

Bonds	Bond length (Å)		ρ_{BCP} (a.u.)		$\nabla^2\rho_{BCP}$		G_{BCP}		V_{BCP}		H_{BCP}		BE_{BCP} (kcal/mol)	
	EXC	HYB	EXC	HYB	EXC	HYB	EXC	HYB	EXC	HYB	EXC	HYB	EXC	HYB
KOH+EG (1:1)														
H-bonds														
O12H6	2.1450		0.0204		0.0596		0.0137		-0.0125		0.0012		-3.8085	
O12H9		1.6230		0.0577		0.1421		0.0463		-0.0571		-0.0108		-12.1294
O12H10		1.5090		0.0767		0.1495		0.0602		-0.0830		-0.0228		-16.3679
Van der Waals interactions														
O1K11	2.7180		0.0164		0.0778		0.0164		-0.0134		0.0030		-2.9162	
O12K11	2.2710		0.0433		0.2528		0.0570		-0.0508		0.0062		-8.9171	
O2K11		2.6080		0.0219		0.1051		0.0226		-0.0189		0.0037		-4.1432
KOH+EG (1:2)														
H-bonds														
O2H20	1.7920	1.7810	0.0376	0.0390	0.1120	0.120	0.0313	0.032	-0.0326	-0.0330	-0.0013	-0.002	-7.6455	-7.9578
O22H9	1.6410	1.6160	0.0564	0.0583	0.1447	0.1503	0.0461	0.0483	-0.0560	-0.0590	-0.0099	-0.0107	-11.8394	-12.2633
O22H10	1.6580	1.6030	0.0527	0.0595	0.1372	0.1474	0.0426	0.0486	-0.0509	-0.0603	-0.0083	-0.0118	-11.0140	-12.5310
O22H19	1.7280	1.6100	0.0449	0.0574	0.1273	0.0151	0.0363	0.0479	-0.0408	-0.0580	-0.0045	-0.0101	-9.2740	-12.0625
Van der Waals interactions														
O1K21	2.7480	2.8000	0.0163	0.0149	0.0724	0.0626	0.0155	0.0135	-0.0128	-0.0114	0.0026	0.0022	-2.8939	-2.5816
O11K21	2.6460	2.7300	0.0210	0.0180	0.0937	0.0761	0.0204	0.0166	-0.0174	-0.0142	0.0030	0.0024	-3.9424	-3.2731
O12K21	2.7570	2.9040	0.0168	0.0122	0.0702	0.0494	0.0153	0.0106	-0.0131	-0.0089	0.0022	0.0017	-3.0054	-1.9793
O22K21	2.7000		0.0172		0.0819		0.0175		-0.0145		0.0030		-3.0947	
KOH+EG (1:4)														
H-bonds														
O11H39	1.9190		0.0273		0.0979		0.0229		-0.0214		0.0015		-5.3478	
O12H30	1.8080		0.0331		0.1173		0.0287		-0.0281		0.0006		-6.6417	
O22H9	1.8630		0.0304		0.1067		0.0256		-0.0246		0.0011		-6.0393	
O21H17	2.4200		0.0108		0.0330		0.0073		-0.0064		0.0009		-1.6670	
O31H20	1.8260		0.0324		0.1119		0.0276		-0.0271		0.0004		-6.4855	
O42H7	2.2860		0.0158		0.0441		0.0100		-0.0092		0.0009		-2.7824	
O42H36	2.1770		0.0190		0.0548		0.0126		-0.0114		0.0011		-3.4962	
O1H16		2.6470		0.0066		0.0200		0.0044		-0.0038		0.0006		-0.7300

O21H40		1.8630		0.0308		0.1064		0.0259		-0.0251		0.0008		-6.1286
O32H9		1.8520		0.0302		0.1095		0.0260		-0.0247		0.0013		-5.9947
O32H30		1.9040		0.0280		0.0960		0.0228		-0.0216		0.0012		-5.5039
**Van der Waals interactions**														
O31H8	2.7790		0.0052		0.0172		0.0037		-0.0031		0.0006		-0.4177	
O2K41	2.6790		0.0176		0.0857		0.0180		-0.0147		0.0034		-3.1839	
O32K41	2.7510		0.0166		0.0722		0.0156		-0.0130		0.0025		-2.9608	
K41O42	2.3490		0.0370		0.2056		0.0457		-0.0400		0.0057		-7.5117	
O11K41		2.7720		0.0149		0.0027		0.0144		-0.0118		0.0027		-2.5816
O22K41		2.7610		0.0157		0.0709		0.0151		-0.0125		0.0026		-2.7601
K41O42		2.4630		0.0272		0.1536		0.0329		-0.0273		0.0056		-5.3255
H36H8		2.7220		0.0023		0.0070		0.0014		-0.0011		0.0004		0.2292
